# Maternal perinatal anxiety and infant primary care use in 1998–2016: a UK cohort study

**DOI:** 10.1136/bmjment-2024-301160

**Published:** 2025-01-09

**Authors:** Holly Christina Smith, Charlotte Archer, James Bailey, Carolyn Chew Graham, Jonathan Evans, Tamsin Fisher, David Kessler, Tom Kingstone, Janine Procter, Noureen Shivji, Victoria Silverwood, Amy Spruce, Katrina Turner, Pensée Wu, Dahai Yu, Irene Petersen

**Affiliations:** 1 Department of Primary Care and Population Health, University College London, London, UK; 2 Population Health Sciences, University of Bristol Medical School, Bristol, UK; 3 School of Medicine, Keele University, Keele, Staffordshire, UK; 4 University of Bristol, Bristol, UK; 5 Just Family CIC, Staffordshire, UK; 6 North Staffordshire Combined Healthcare NHS Trust, Trentham, Staffordshire, UK

**Keywords:** Anxiety disorders, Data Interpretation, Statistical

## Abstract

**Background:**

There is some evidence that perinatal anxiety (PNA) is associated with lower rates of infant vaccinations and decreased access to preventative infant healthcare, but results across studies have not been conclusive.

**Objective:**

To investigate the relationship between maternal PNA and infant primary care use.

**Methods:**

Cohort study of mother-infant pairs identified between 1998 and 2016 using IQVIA Medical Research Database (IMRD). PNA was identified through prescription, diagnosis and symptom records from start of pregnancy up to 1 year after birth. Outcomes include primary care consultation rate, attendance at the 6–8 week infant check and uptake of the infant 5-in-1 vaccination, comparing unadjusted rates of consultations and using logistic regression to analyse other outcomes.

**Findings:**

Of the 248 618 women, 11 558 (4.7%) had a record of PNA. Infants of mothers with PNA had, on average, one more primary care consultation/person-year compared with those without (9.7 vs 8.7) in the year after birth. Mothers with PNA were more likely to have an infant who was vaccinated (adjusted OR (aOR) 1.33, 95% CI 1.20 to 1.48) but were less likely to have a record of attendance at the 6–8 week infant check (aOR 0.88, 95% CI 0.81 to 0.95).

**Conclusions:**

Infants of mothers with PNA had, on average, a slightly higher rate of primary care consultations and were more likely to be vaccinated but less likely to have a record of an infant check.

**Clinical implications:**

Midwives and General Practitioners (GPs) providing care should consider how PNA may impact on infant health and how infant health may impact on maternal anxiety.

**WHAT IS ALREADY KNOWN ON THIS TOPIC**

- To date, there has been no large UK study assessing/identifying the relationship directly between perinatal anxiety (PNA) and uptake of the infant 5-in-1 vaccination and the 6–8 week infant check; nor do we know what role infant health plays in the relationship between PNA and infant healthcare use.

**WHAT THIS STUDY ADDS**

- We found that infants of mothers with PNA were more likely to be vaccinated than those without PNA; however, we found that these infants were less likely to have a record of a 6–8 week infant check and have slightly higher primary care consultation rates, which needs further consideration.
- While we were limited by missing data in this study, our analysis suggests that infant health may at least partly account for the higher consultation rate in infants of mothers with PNA.

**HOW MIGHT THIS STUDY AFFECT RESEARCH, PRACTICE OR POLICY**

- Midwives and GPs providing care should carefully consider how PNA may impact on infant health and how infant health may, in turn, relate to anxiety in mothers.

## Background

 Perinatal anxiety (PNA) is a common perinatal mental illness and can have a negative impact on mothers, their children and society.^1^ It is estimated that around 15.2% of women are diagnosed with PNA during pregnancy and around 9.9% are diagnosed in the year after childbirth.^2^ Risk factors include the lack of a partner or social support, history of abuse, domestic violence or mental illness, unwanted or unplanned pregnancy, adverse life events, high perceived stress, present/past pregnancy complications, pregnancy loss and having a medically complex pregnancy.^1^ There is some evidence that PNA is associated with lower rates of infant vaccinations and decreased access to preventative infant healthcare,^3 4^ but the results across studies have not been conclusive.^5^

In the UK, the main vaccination given to children is the 5-in-1 vaccine. It was introduced in 2006 and was replaced by the 6-in-1 vaccine in 2017, prior to this, these vaccinations were offered separately.^6^ This combined vaccination protects against diphtheria, tetanus, pertussis (whooping cough), polio and Hib disease (Haemophilus influenzae type b). UK vaccination rates are typically high and in line with the WHO target of 95% of children.^7^ Infants should receive three doses of the 5-in-1 vaccination, scheduled at weeks 8, 12 and 16 after birth. To allow for flexibility in the timing of these doses, national coverage to receive all three doses is measured at 12 months after birth.^8^ The largest UK-based study of nearly 500 000 mother-infant pairs found that, between 1993 and 2015, infants of mothers with an anxiety disorder were 14% less likely to be fully vaccinated by 2 years old compared with mothers with no anxiety.^4^ While authors found lower rates in those with an anxiety disorder, they did not distinguish between uptake of the mumps, measles and rubella (MMR) and the 5-in-1 vaccine in their outcome. As such, it is not clear how the historic controversies and low uptake of the MMR vaccine in the 1990s and 2000s^9^ impact on the relationship between anxiety and infant vaccinations in a large UK cohort.

There is also some evidence that PNA is associated with increased overall healthcare use in infants,^5 10 11^ but again this has not been a consistent finding across studies.^12 13^ One large UK study of ~500 000 children found that maternal mental illness (including PNA) was associated with increased infant healthcare use^11^; however, authors did not present results for PNA alone, making it challenging to draw conclusions. We found no previous studies exploring PNA and uptake of the 6–8 week infant check. The 6–8 week infant check takes place in primary care, usually performed by a General Practitioner (GP) and is a ‘top-to-toe’ examination which includes checking infant’s eyes, heart, hips and genitalia, alongside measuring length, weight and head circumference.^14^ It often takes place at the same time as the mother’s postnatal check^15^ and the infant’s vaccinations. While these studies indicate there may be a relationship between PNA and infant healthcare use, their findings are not conclusive across studies and have some limitations. To date, there has been no large UK study assessing/identifying the relationship directly between PNA and uptake of the infant 5-in-1 vaccination and the 6–8 week infant check. In addition, it is not clear what role infant health plays in the relationship between PNA and infant healthcare use.

## Objective

The aim of this study was to use linked mother and infant primary care electronic health records to investigate if infants were less likely to receive planned preventative care after birth if their mother had a record of PNA. We also explored overall infant primary care use in these groups and how birth/infant characteristics and antenatal or postnatal anxiety impacts this relationship.

## Methods

### Data source

We used a UK primary care database, IQVIA Medical Research Database (IMRD). IQVIA Medical Research Data (IMRD) incorporates data from THIN, A Cegedim Database. Reference made to THIN is intended to be descriptive of the data asset licensed by IQVIA. This work uses deidentified data provided by patients as a part of their routine primary care. As of December 2016, IMRD contained anonymised electronic health records for 16 million registered patients from 730 UK practices^16^ and contains patient-level information on demographics, prescribing, diagnoses/symptoms and preventative healthcare measures, including vaccinations. Diagnoses/symptoms are recorded using Read codes^17^ and vaccinations are recorded using Additional Health Data (AHD) records. Prescribing information is categorised according to British National Formulary (BNF) codes.^18^

### Study population

#### Mother-infant cohort

We included women of childbearing potential (aged 15–49 years) whose pregnancy started between 1 January 1998 and 31 December 2016. We included women who had been registered at a practice for at least 2 years before start of pregnancy. Infants and/or mothers with less than 1 year of follow-up information after the date of childbirth (eg, died or transferred practice) were also excluded. Potential mother-infant pairs were identified by a recorded childbirth in a women’s record and an infant first registered within the same household at the time of birth. In a mother’s record, childbirth and date were determined using a combination of an antenatal record, delivery record, postnatal care record or date of last menstrual period using a pre-existing algorithm.^19^ If women had multiple infants in the study period, one was selected at random.

### Definition of variables

#### Maternal perinatal anxiety

Women were followed up throughout pregnancy and up to 12 months after the date of childbirth—the perinatal period. Mothers were defined as having PNA if their records contained a recorded symptom or a diagnosis of anxiety or a prescription for an anxiolytic drug (including selective serotonin reuptake inhibitors (SSRIs)). For SSRI prescriptions, we excluded women who were identified as having PNA ONLY through an SSRI prescription (eg, no diagnostic/symptom code or no prescription for another anxiolytic) AND where they had a symptom or diagnostic code indicating depression either in the perinatal period and/or in 2 years prior to start of pregnancy. We also stratified PNA into antenatal anxiety and postnatal anxiety. Women could have both antenatal and postnatal anxiety.

Sensitivity analyses considered mothers defined as having PNA if their records contained at least one of either: a recorded symptom or diagnosis of anxiety (as above) or a prescription for an anxiolytic drug (including SSRIs) in the perinatal period, with no exclusions in relation to prescribing SSRIs for depression.

Additional exclusions were applied to the main analysis and sensitivity analysis whereby an anxiolytic drug or SSRI may have been prescribed for something other than anxiety, for example, a benzodiazepine prescribed for epilepsy or muscle spasticity. All exclusions alongside full drug, symptom and diagnostics code lists are included in the online supplemental material.

#### Outcome: infant healthcare utilisation

Healthcare utilisation was defined as the number of primary care consultations infants had recorded in the first year after birth. Primary care consultations were defined as any direct encounters (at home, in the clinic or by telephone) for each infant in the year after birth. Multiple encounters on a single day were grouped for a maximum of one primary care consultation per infant per day. The location of the encounters was also reported. Where there were multiple entries for each day but with different locations, these were assigned by a hierarchy of: clinic, home and telephone.

#### Outcome: infant health check

We identified the 6–8 week infant check through an infant’s AHD record and/or their medical record. A code list identifying this check is included in the supplemental appendix. Recording of 6–8 week infant health checks has been consistent from 2001 onwards, therefore, we only include data from 2001 onwards for this outcome.

#### Outcome: infant vaccination adherence

Our outcome was three doses of the 5-in-1 vaccine in infants between date of birth and up to 1 year after birth. The 5-in-1 vaccine was identified through an infant’s AHD record. While our study period starts before the individual vaccinations were offered as a combined dose (from 2006 onwards), after exploring our data from before this, we found that these vaccinations are recorded consistently in our data source under a combined code for the entire study period with similar rates of vaccination in each year. Thus, we chose to include outcome information on infant vaccinations for the entire study period.

#### Maternal or infant characteristics

Analyses were stratified by maternal and infant characteristics. Maternal characteristics included age at childbirth (years), Townsend score (a measure of social deprivation), history of anxiety in the 2 years prior to pregnancy, history of depression in the 2 years prior to pregnancy, mode of delivery and calendar year. Maternal age was grouped into 5-year bands. Calendar year was grouped into 2-year bands. Infant characteristics included sex of the infant, gestation at delivery (in weeks), neonatal intensive care unit (NICU) admissions at birth, Appearance, Pulse, Grimace, Activity and Respiration (APGAR) score (a measure used to evaluate the health of a newborn with a score between 0 and 10) at 1 and 5 min after birth and infant birth weight.

### Statistical analysis

All analyses were conducted using Stata V.16 (StataCorp, College Station, Texas, USA). For healthcare utilisation, unadjusted rates of primary care consultations were calculated as the total number of consultations over the number of person-years, stratified by maternal and infant characteristics. To examine uptake of the 5-in-1 vaccine and attendance at the 6–8 week infant check, we calculated the unadjusted proportion of those with the outcome and then we used Logistic regression models for adjusted estimates. Three models were constructed: unadjusted, age-adjusted and age-deprivation-year adjusted. To account for clustering by general practice, GP practice was included as a random effects term (both random slopes and intercepts at the practice level) in these models.

## Findings

### Participants

**Figure 1:**
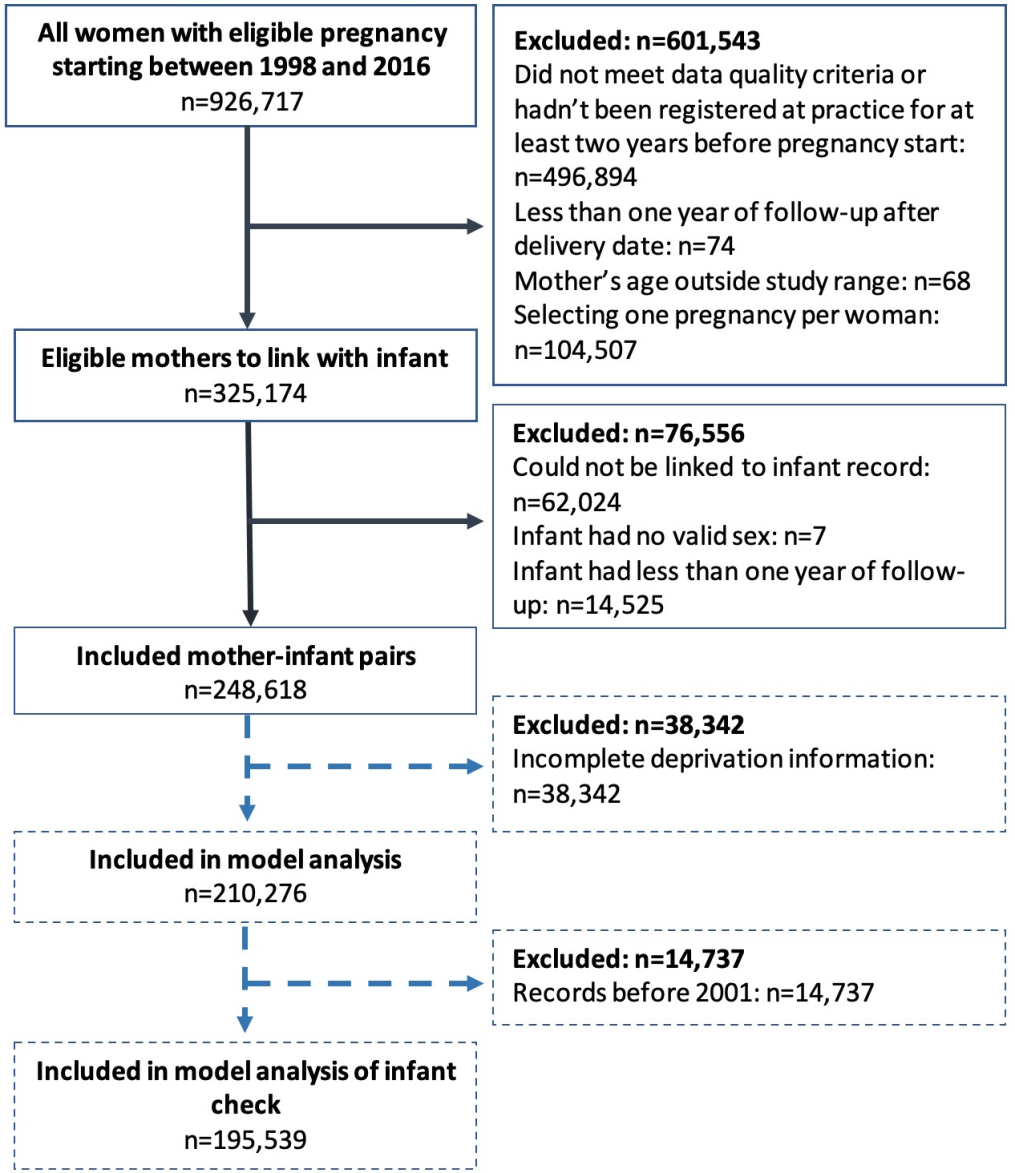
Flow diagram showing application of study inclusion and exclusion criteria.

We identified 248 618 mother-infant pairs where the pregnancy started between 1 January 1998 and 31 December 2016 (figure 1).

### Maternal and infant characteristics

Of the women included in this study, 4.7% (n=11 558) had a record of PNA, 2.2% (n=5505) had a record of antenatal anxiety and 3.6% (n=9023) had a record of postnatal anxiety (table 1). A higher proportion of women in younger age groups had PNA (6.5% of those aged 15–19 years and 6.2% of those aged 20–24 years vs 4.0% of those aged 30–34 years) and those from more deprived areas were more likely to have PNA (5.7% in most deprived compared with 3.5% in least deprived). Those with a recent history of anxiety or depression were much more likely to have PNA (31.8% of those with recent anxiety vs 2.6% of those with no history of anxiety and 30.3% of those with recent depression vs 3.0% of those with no history of depression). PNA increased over time from 3.3% in 1998–2001 to 6.6% in 2014–2016. Mothers of infants who were born preterm were more likely to have a record of PNA than those born at term (6.4% of those born before 32 weeks gestation and 5.8% of those born between 32 and 37 weeks vs 3.8% of those born after 37 weeks). Mothers of infants who had a low APGAR score 1 min after birth were more likely to have PNA (7.0% of those with score 0–3 vs 4.1% of those with score 7–10) and mothers whose infants were admitted to NICU were more likely to have PNA (6.9% of those with a record of NICU admission vs 4.6% with no record). Rates of PNA were similar across other variables and trends across characteristics were comparable for antenatal anxiety and postnatal anxiety (table 1).

**Table 1:** Maternal and infant characteristics comparing those with perinatal anxiety, antenatal anxiety and postnatal anxiety

Characteristics	TotalN (% down)	Perinatal anxietyN (% across)	Antenatal anxietyN (% across)	Postnatal anxietyN (% across)
Overall	248 618	11 558 (4.7)	5505 (2.2)	9023 (3.6)
Maternal age (years)				
15–19	8885 (3.6)	576 (6.5)	196 (2.2)	495 (5.6)
20–24	31 069 (12.5)	1939 (6.2)	864 (2.8)	1526 (4.9)
25–29	56 438 (22.7)	2706 (4.8)	1291 (2.3)	2118 (3.8)
30–34	79 810 (32.1)	3168 (4.0)	1544 (1.9)	2470 (3.1)
35–39	56 757 (22.8)	2340 (4.1)	1172 (2.1)	1787 (3.2)
40–44	14 809 (6.0)	782 (5.3)	416 (2.8)	592 (4.0)
45–49	850 (0.3)	47 (5.5)	22 (2.6)	35 (4.1)
Townsend Score quintile				
1—least deprived	49 205 (23.4)	1732 (3.5)	800 (1.6)	1324 (2.7)
2	42 271 (20.1)	1731 (4.1)	817 (1.9)	1322 (3.1)
3	45 729 (21.8)	2171 (4.8)	1037 (2.3)	1692 (3.7)
4	42 101 (20.0)	2172 (5.2)	1060 (2.5)	1694 (4.0)
5—most deprived	30 970 (14.7)	1756 (5.7)	875 (2.8)	1381 (4.5)
Unknown	38 342	1996 (5.2)	916 (2.4)	1610 (4.2)
History of anxiety				
Recent	14 712 (5.9)	4673 (31.8)	3141 (21.4)	3781 (25.7)
Previous	6461 (2.6)	1080 (16.7)	401 (6.2)	826 (12.8)
None	227 445 (91.5)	5805 (2.6)	1963 (0.9)	4416 (1.9)
History of depression				
Recent	12 284 (4.9)	3721 (30.3)	2669 (21.7)	3033 (24.7)
Previous	5085 (2.1)	902 (17.7)	371 (7.3)	714 (14.0)
None	231 249 (93.0)	6935 (3.0)	2465 (1.1)	5276 (2.3)
Mode of delivery				
Vaginal delivery	75 096 (63.9)	2975 (4.0)	1400 (1.9)	2325 (3.1)
Instrumental	15 586 (13.3)	960 (6.2)	457 (2.9)	756 (4.9)
Caesarean	26 821 (22.8)	1415 (5.3)	693 (2.6)	1100 (4.1)
Unknown	131 115	6208 (4.7)	2955 (2.3)	4842 (3.7)
Year group				
1998–2001*	25 345 (10.2)	843 (3.3)	358 (1.4)	665 (2.6)
2002–2004	39 098 (15.7)	1519 (3.9)	705 (1.8)	1161 (3.0)
2005–2007	47 741 (19.2)	1950 (4.1)	878 (1.8)	1504 (3.2)
2008–2010	51 713 (20.8)	2389 (4.6)	1142 (2.2)	1840 (3.6)
2011–2013	50 075 (20.1)	2570 (5.1)	1279 (2.6)	2024 (4.0)
2014–2016	34 646 (13.9)	2287 (6.6)	1143 (3.3)	1829 (5.3)
**Infant characteristics**				
Sex				
Male	126 988 (51.1)	5915 (4.7)	2854 (2.3)	4583 (3.6)
Female	121 630 (48.9)	5643 (4.6)	2651 (2.2)	4440 (3.7)
Gestation at childbirth (weeks)				
<31.9	545 (1.3)	35 (6.4)	16 (2.9)	28 (5.1)
32–36.9	2361 (5.7)	136 (5.8)	72 (3.1)	105 (4.5)
>37	38 474 (93.0)	1465 (3.8)	734 (1.9)	1093 (2.8)
Unknown	207 238	9922 (4.8)	4683 (2.3)	7797 (3.8)
Birth weight (kg)				
<1.50	24 (0.4)	–	–	–
1.50–2.49	233 (4.1)	12 (5.2)	8 (3.4)	7 (3.0)
2.50–2.99	838 (14.8)	41 (4.9)	26 (3.1)	32 (3.8)
3.00–3.49	2022 (35.8)	85 (4.2)	41 (2.0)	61 (3.0)
3.50–3.99	1798 (31.8)	64 (3.6)	33 (1.8)	48 (2.7)
>4.00	739 (13.1)	26 (3.5)	8 (1.1)	20 (2.7)
Unknown	242 964	11 327 (4.7)	5386 (2.2)	8853 (3.6)
APGAR at 1 min				
0–3	588 (1.7)	41 (7.0)	22 (3.7)	38 (6.5)
4–6	2043 (6.0)	88 (4.3)	47 (2.3)	65 (3.2)
7–10	31 702 (92.3)	1286 (4.1)	651 (2.1)	940 (3.0)
Unknown	214 285	10 143 (4.7)	4785 (2.2)	7980 (3.7)
APGAR at 5 min				
0–3	65 (0.2)	–	–	–
4–6	377 (1.1)	21 (5.6)	10 (2.7)	16 (4.2)
7–10	33 665 (98.7)	1384 (4.1)	708 (2.1)	1014 (3.0)
Unknown	214 511	10 149 (4.7)	4786 (2.2)	7989 (3.7)
Special care at birth				
NICU	6477 (2.6)	449 (6.9)	201 (3.1)	369 (5.7)
Unknown	242 141 (97.4)	11 109 (4.6)	5304 (2.2)	8654 (3.6)

Categories labelled as ‘unknown’ include missing data and those where it is not possible to determine a value.

* This group is larger than the others.

APGARAppearance, Pulse, Grimace, Activity and RespirationNICUneonatal intensive care unit

### Infant healthcare use

Overall, infants of mothers with PNA had 1.0 more primary care consultation/person-year in the year after birth compared with those without PNA (9.7 vs 8.7) (table 2). This was similar for those with antenatal (9.8 consultations/person-year) and postnatal (9.7 consultations/person-year) anxiety. There was little variation in primary care consultation rates across maternal characteristics (such as maternal age or deprivation) for those both with and without PNA; however, rates did vary over time and for some infant characteristics. Among infants of mothers with PNA consultation rates decreased over time, from 10.3 (10.1–10.5) per person-year in 1998–2001 to 9.1 (9.0–9.2) per person-year in 2014–2016. Among infants of mothers with PNA, consultation rates were higher for those born between 32 and 37 weeks gestation (12.1 consultations/person-year), those with a birth weight between 1.50 and 2.49 kg (12.4 consultations/person-year) and those with low APGAR scores at 1 min after birth (11.3 consultations/person-year for those with a score 4–6). These trends were similar among infants whose mothers did not have PNA and for those antenatal and/or postnatal depression (table 2). Most infant consultations (93.1%) took place in the clinic and there was a slightly higher proportion of phone consultations for infants whose mother had PNA compared with no PNA (6.0% vs 4.9%) (data not shown).

**Table 2:** Infant healthcare use, comparing those with and without maternal perinatal anxiety, antenatal anxiety and postnatal anxiety

Characteristics	Rate of consultations per person-year (95% CIs)			
	No anxiety	Perinatal anxiety	Antenatal anxiety	Postnatal anxiety
Overall	8.7 (8.7 to 8.7)	9.7 (9.6 to 9.8)	9.8 (9.7 to 9.9)	9.7 (9.6 to 9.8)
Maternal age (years)				
15–19	8.8 (8.8 to 8.9)	9.5 (9.3 to 9.8)	10.0 (9.5 to 10.4)	9.5 (9.3 to 9.8)
20–24	9.0 (9.0 to 9.0)	9.9 (9.7 to 10.0)	10.3 (10.0 to 10.5)	9.9 (9.7 to 10.1)
25–29	8.9 (8.9 to 9.0)	10.0 (9.9 to 10.1)	10.0 (9.8 to 10.2)	10.0 (9.9 to 10.2)
30–34	8.7 (8.7 to 8.7)	9.7 (9.6 to 9.8)	9.8 (9.7 to 10.0)	9.7 (9.6 to 9.8)
35–39	8.3 (8.3 to 8.4)	9.3 (9.2 to 9.5)	9.3 (9.1 to 9.5)	9.3 (9.2 to 9.5)
40–44	8.0 (8.0 to 8.1)	9.3 (9.1 to 9.6)	9.6 (9.3 to 9.9)	9.2 (9.0 to 9.5)
45–49	8.2 (8.0 to 8.4)	8.4 (7.6 to 9.2)	7.8 (6.7 to 9.0)	8.3 (7.4 to 9.3)
Townsend Score quintile				
1—least deprived	8.8 (8.8 to 8.8)	10.0 (9.8 to 10.1)	10.0 (9.8 to 10.2)	10.0 (9.8 to 10.1)
2	8.7 (8.6 to 8.7)	9.9 (9.8 to 10.0)	10.0 (9.8 to 10.0)	10.0 (9.8 to 10.2)
3	8.7 (8.7 to 8.7)	9.7 (9.6 to 9.8)	9.8 (9.6 to 10.0)	9.7 (9.5 to 9.8)
4	8.8 (8.8 to 8.9)	9.7 (9.6 to 9.8)	9.8 (9.6 to 10.0)	9.8 (9.6 to 9.9)
5—most deprived	8.7 (8.7 to 8.8)	9.6 (9.5 to 9.8)	9.7 (9.5 to 10.0)	9.6 (9.4 to 9.8)
Unknown	8.3 (8.3 to 8.3)	9.3 (9.2 to 9.5)	9.5 (9.3 to 9.7)	9.3 (9.1 to 9.4)
History of anxiety				
Recent	9.3 (9.2 to 9.4)	9.8 (9.7 to 9.9)	9.8 (9.7 to 9.9)	9.9 (9.8 to 10.0)
Previous	9.3 (9.2 to 9.4)	9.9 (9.7 to 10.1)	10.4 (10.1 to 10.7)	9.8 (9.6 to 10.0)
None	8.6 (8.6 to 8.6)	9.6 (9.5 to 9.6)	9.8 (9.6 to 9.9)	9.5 (9.4 to 9.6)
History of depression				
Recent	9.2 (9.1 to 9.3)	9.7 (9.6 to 9.8)	9.6 (9.5 to 9.8)	9.8 (9.7 to 9.9)
Previous	9.2 (9.1 to 9.3)	10.0 (9.8 to 10.2)	10.5 (10.1 to 10.8)	9.9 (9.7 to 10.2)
None	8.6 (8.6 to 8.7)	9.7 (9.6 to 9.7)	9.9 (9.8 to 10.0)	9.6 (9.5 to 9.7)
Mode of delivery				
Vaginal delivery	8.7 (8.7 to 8.7)	9.6 (9.5 to 9.8)	9.9 (9.7 to 10.0)	9.6 (9.5 to 9.7)
Instrumental	9.2 (9.1 to 9.2)	10.0 (9.8 to 10.2)	10.0 (9.7 to 10.3)	10.1 (9.9 to 10.3)
Caesarean	8.8 (8.8 to 8.9)	9.9 (9.7 to 10.0)	10.2 (9.9 to 10.4)	9.9 (9.7 to 10.0)
Unknown	8.6 (8.5 to 8.6)	9.6 (9.6 to 9.7)	9.7 (9.6 to 9.8)	9.6 (9.6 to 9.7)
Year group				
1998–2001*	9.2 (9.2 to 9.2)	10.3 (10.1 to 10.5)	10.6 (10.2 to 10.9)	10.3 (10.1 to 10.6)
2002–2004	8.8 (8.8 to 8.9)	10.0 (9.8 to 10.1)	10.2 (10.0 to 10.5)	10.0 (9.8 to 10.2)
2005–2007	8.9 (8.9 to 9.0)	10.1 (9.9 to 10.2)	10.3 (10.1 to 10.6)	10.0 (9.9 to 10.2)
2008–2010	8.7 (8.6 to 8.7)	9.6 (9.5 to 9.8)	9.7 (9.5 to 9.9)	9.6 (9.4 to 9.7)
2011–2013	8.6 (8.5 to 8.6)	9.6 (9.5 to 9.8)	9.8 (9.7 to 10.0)	9.6 (9.5 to 9.8)
2014–2016	7.9 (7.8 to 7.9)	9.1 (9.0 to 9.2)	9.0 (8.8 to 9.2)	9.2 (9.1 to 9.3)
**Infant characteristics**				
Sex				
Male	9.0 (8.9 to 9.0)	9.9 (9.8 to 10.0)	10.1 (9.9 to 10.2)	9.9 (9.8 to 10.0)
Female	8.4 (8.3 to 8.4)	9.5 (9.4 to 9.5)	9.6 (9.4 to 9.7)	9.5 (9.4 to 9.6)
Gestation at childbirth (weeks)				
<31.9	9.8 (9.5 to 10.1)	8.9 (8.0 to 10.0)	10.0 (8.5 to 11.7)	8.7 (7.6 to 9.9)
32–36.9	10.4 (10.2 to 10.5)	12.1 (11.5 to 12.7)	11.8 (11.0 to 12.6)	12.3 (11.6 to 13.0)
>37	9.5 (9.4 to 9.5)	10.9 (10.7 to 11.0)	11.1 (10.8 to 11.3)	11.1 (10.9 to 11.3)
Unknown	8.5 (8.5 to 8.5)	9.5 (9.4 to 9.6)	9.6 (9.5 to 9.7)	9.5 (9.4 to 9.5)
Birth weight (kg)				
<1.50	11.2 (9.8 to 12.8)	–	–	–
1.50–2.49	9.9 (9.5 to 10.3)	12.4 (10.5 to 14.6)	13.6 (11.2 to 16.4)	12.3 (9.8 to 15.2)
2.50–2.99	9.0 (8.8 to 9.3)	10.3 (9.3 to 11.3)	10.2 (9.0 to 11.5)	10.4 (9.3 to 11.6)
3.00–3.49	9.0 (8.9 to 9.2)	9.8 (9.2 to 10.5)	9.0 (8.1 to 9.9)	9.8 (9.0 to 10.6)
3.50–3.99	9.1 (8.9 to 9.2)	10.3 (9.5 to 11.1)	9.4 (8.4 to 10.6)	10.6 (9.7 to 11.6)
>4.00	8.7 (8.5 to 8.9)	10.2 (9.0 to 11.5)	8.4 (6.5 to 10.6)	10.5 (9.1 to 12.0)
Unknown	8.7 (8.6 to 8.7)	9.7 (9.6 to 9.7)	9.8 (9.7 to 9.9)	9.7 (9.6 to 9.8)
APGAR at 1 min				
0–3	10.0 (9.8 to 10.3)	–	–	–
4–6	10.1 (9.9 to 10.2)	11.3 (10.6 to 12.1)	11.8 (10.8 to 12.9)	11.2 (10.3 to 12.0)
7–10	9.4 (9.4 to 9.5)	11.0 (10.8 to 11.2)	11.2 (11.0 to 11.5)	11.2 (10.9 to 11.4)
Unknown	8.5 (8.5 to 8.6)	9.5 (9.5 to 9.6)	9.6 (9.5 to 9.7)	9.5 (9.4 to 9.6)
APGAR at 5 min				
0–3	9.9 (9.2 to 10.8)	–	–	–
4–6	10.1 (9.8 to 10.4)	11.9 (10.4 to 13.5)	10.9 (8.8 to 13.3)	12.0 (10.4 to 13.8)
7–10	9.5 (9.5 to 9.5)	11.1 (10.9 to 11.3)	11.3 (11.1 to 11.6)	11.2 (11.0 to 11.4)
Unknown	8.5 (8.5 to 8.5)	9.5 (9.4 to 9.6)	9.6 (9.5 to 9.7)	9.5 (9.4 to 9.6)
Special care at birth				
NICU	9.0 (9.0 to 9.1)	9.8 (9.5 to 10.1)	9.8 (9.3 to 10.2)	9.8 (9.5 to 10.2)
Unknown	8.7 (8.6 to 8.7)	9.7 (9.6 to 9.8)	9.8 (9.7 to 9.9)	9.7 (9.6 to 9.8)

*This group is larger than the others.

APGARAppearance, Pulse, Grimace, Activity and RespirationNICUneonatal intensive care unit

### Infant 5-in-1 vaccination adherence and attendance at 6–8 week infant check

Overall vaccination rate in this study was high (94.8%) and was similar for infants of mothers with and without PNA in unadjusted analysis (94.7% vs 95.3%) (table 3). However, once we adjusted for maternal age, deprivation and year, infants of mothers with PNA were 33% (95% CI 20% to 48%) more likely to have their 5-in-1 vaccination. In terms of the 6–8 week infant check, in unadjusted analysis, the proportion with a record of a check was broadly similar for infants of mothers with and without PNA (80.7% vs 82.7%). However, once adjusted for maternal age, deprivation and year, infants of mothers with PNA were 12% (95% CI 19% to 5%) less likely to have a record of a 6–8 week infant check compared with those with no PNA (table 3).

**Table 3:** Infant preventative healthcare uptake and model analysis, comparing those with and without maternal perinatal anxiety

Outcomes	5-in-1 vaccination		6–8 week infant check*	
	Yes	No	Yes	No
**n (%)**				
No perinatal anxiety	224 583 (94.7)	12 477 (5.3)	183 051 (82.7)	38 271 (17.3)
Perinatal anxiety	11 018 (95.3)	540 (4.7)	8899 (80.7)	2134 (19.3)
**Model analysis**	**Unadjusted: OR (95% CI)**	**Adjusted†** **: OR (95% CI)**	**Unadjusted: OR (95% CI)**	**Adjusted†** **: OR (95% CI)**
No perinatal anxiety	1	1	1	1
Perinatal anxiety	1.15 (1.04–1.28)	1.33 (1.20–1.48)	0.89 (0.82–0.97)	0.88 (0.81–0.95)

* Analysis includes records from 2001 onwards only (n=232 355 for summary statistics and n=195 539 for model analysis).

† Adjusted for age, deprivation and year. Practice is included as a random effects term in all models.

### Sensitivity analysis

We observed similar trends across characteristics when we used a more sensitive approach to identify PNA but there was a higher proportion of women had PNA (8.6%) (online supplemental table A-C).

## Discussion

### Main findings

We found that primary care healthcare use was slightly higher in infants of mothers who experienced PNA compared with those with no PNA in their first year after birth (9.7 vs 8.7 primary care consultations/person-year). In all women, regardless of PNA, we found that primary care consultation rates were higher for infants born prematurely, those with a low birth weight and those with low APGAR scores. We found no difference in infant vaccination uptake between mothers with and without PNA (94.7% vs 95.3%) in unadjusted analysis. However, once adjusted for maternal age, deprivation and year, mothers with PNA were 33.0% more likely to have an infant who received their 5-in-1 vaccination. We found a small difference in unadjusted analysis of the 6–8 week infant check-up when comparing mothers with and without PNA (80.7% vs 82.7%). However, in adjusted analysis, those with PNA were 12% less likely to have a record of a 6–8 week infant check compared with those without. PNA was more common in younger women, those living in more deprived areas, those with a recent history of anxiety or depression, those who had a premature birth, and those whose infants were admitted to NICU or had a low APGAR score at birth.

### Study strengths and limitations

This study used a large population-based cohort (n=248 618 mother-infant pairs) to investigate PNA and infant healthcare use in UK general practice.

We were limited by what was recorded in electronic health records and by missing data, in particular for measures relating to infant health. For example, while we found similar uptake of the infant vaccination, we did find a difference in uptake of the infant check. As the infant check often happens at the same time as the 5-in-1 vaccination is administered, it is likely a higher proportion of infants in this study received a 6–8 week infant check in reality, but it was not documented in their electronic health record. Since 2020/2021, GPs have been contractually obligated to perform the 6–8 week check and this needs to be recorded in primary care records for GPs to receive payment.^20^ We anticipate that this change would lead to improved documentation of this check, but this could be explored in future studies. We also acknowledge that it can be challenging to distinguish between feeling anxious (a common feeling for pregnant women/mothers) and experiencing an anxiety disorder and it is difficult to untangle this in our data. It is likely that we are capturing more severe cases of anxiety, as to be identified in our study required women to seek medical help for their anxiety, report it to a healthcare professional and have it documented in their records. As such, we likely are underestimating the total burden of perinatal anxiety, as not all pregnant/postpartum women may disclose anxious thoughts or feelings. This may be shown in our relatively low estimate of PNA (4.7%). We were also not able to adjust for all potential confounders which may impact the relationship between PNA and infant healthcare use, such as maternal education level, marital/cohabiting status and comorbidities, these could be explored in future studies. Lastly, we had to make assumptions surrounding SSRI prescriptions as they can be prescribed for both depression and anxiety. If there is no related symptom or diagnostic code detailed in a woman’s record, it can be challenging to attribute this prescription to either anxiety, depression or both. To explore this, we conducted a sensitivity analysis which included all SSRI prescriptions, regardless of a diagnosis/symptom indicating depression and this assumed they were prescribed to treat anxiety. Our results were comparable to the main analysis.

### Findings in relation to previous studies

Our estimate of PNA (4.7%) is lower than that identified in previous studies. A 2017 systematic review estimated that 15.2% of women were diagnosed with anxiety during pregnancy and 9.3–9.9% of women were diagnosed in the year after childbirth.^2^ Our lower estimate is likely due to differences in the way anxiety is recorded, as authors note the majority of studies included in their review identified anxiety through self-reported measures, which are likely to provide higher estimates than those captured through electronic health records.

Previous studies exploring the relationship between PNA and infant healthcare use have found varied results between studies. Similar to our findings, the largest previous UK study of roughly 500 000 children found that maternal mental illness, which included anxiety, was associated with increased healthcare use.^11^ The largest UK-based study of nearly 500 000 mother-infant pairs found that infants of mothers with an anxiety disorder were less likely to be fully vaccinated (MMR and 5-in-1) by 2 years old.^4^ In contrast, we found infants of mothers with PNA were more likely to be vaccinated than those with no anxiety. This difference is likely due to our more restrictive outcome measure and follow-up period.

### Explanation of findings

Overall, our findings across outcome measures presents a mixed picture. It is reassuring that infants of mothers with PNA were more likely to be vaccinated than those with no anxiety; however, we found that these infants were less likely to have a record of a 6–8 week check and have slightly higher primary care consultation rates which needs further consideration. It is important to explore if differences in the 6–8 week infant check are due to limitations with data recording or if infants are genuinely missing out on this important appointment. Alongside the higher consultation rate for those whose mother had PNA, we also found higher consultation rates for infants born prematurely, those with a low birth weight and those with low APGAR scores. All of these characteristics were more common in the PNA group, suggesting that higher infant healthcare use may be, at least in part, related to the health of the infant and not necessarily anxiety in the mother. While we are limited by missing data for these key variables in this study, it is essential that future research includes measures on infant health when investigating the relationship between PNA and infant healthcare use. This could be further explored, for example, through linked maternity and primary care data.

### Clinical implications

GPs, midwives and health visitors should be vigilant for signs of PNA in mothers, especially if a child or infant is attending more medical appointments than expected. These healthcare professionals should take the opportunity to identify and support women who may be at higher risk of PNA. This includes younger women, those from more deprived areas, those with a recent history of anxiety or depression, mothers of premature infants, and those whose infants were admitted to the NICU or had a low APGAR score at birth. Key birth factors and the infant’s health should also be discussed with mothers during their 6–8 week postnatal check-up, particularly regarding how these factors might affect their overall health and recovery after childbirth. Although differences in attendance at the infant 6–8 week check-up identified in our study may be due to data recording issues, clinicians should ensure all infants attend this critical appointment. If any infants miss this check-up, follow-up is essential, as most infants will be visiting primary care for vaccinations around this time.

## supplementary material

10.1136/bmjment-2024-301160 online supplemental file 1

## Data Availability

Data may be obtained from a third party and are not publicly available.
