# Supplementary material for: Maternal perinatal anxiety and infant primary care use in 1998–2016: a UK cohort study
Source: BMJ Ment Health. 2025 Jan 9;28(1):e301160. doi: 10.1136/bmjment-2024-301160 (PMC11752066; doi:10.1136/bmjment-2024-301160)
Supplement: online supplemental file 1 [file bmjment-28-1-s001.pdf]

Appendix
Exclusions:

For inclusion into the study, practices that did not meet our data quality criteria of acceptable computer use (ACU) or acceptable mortality rates (AMR) by the start of pregnancy were excluded. ACU is the date a practice was continuously entering on average at least two therapy records, one medical record and one additional health data record per patient per year (a) and AMR is the date a practice has comparable mortality rates to the rest of the UK, given the size and demographics of the practice (b).

PNA exclusions:

Those identified as having PNA through a benzodiazepine prescription in the perinatal period only but had a record of having epilepsy and/or muscle spasticity, and/or a record of alcohol withdrawal in the two years before start of pregnancy up to the end of the perinatal period were considered not to have perinatal anxiety.

Those identified as having PNA through an antipsychotic prescription in the perinatal period only but also had a record of psychosis in the five years before the start of pregnancy up to the end of the perinatal period were considered not to have perinatal anxiety.

Those identified as having PNA through a beta-blocker prescription in the perinatal period only but had a record of cardiovascular event and/or a neurological event in the two years before start of pregnancy up to the end of the perinatal period were considered not to have perinatal anxiety.

All codes lists for these exclusions are included in the appendix below.

(a) Horsfall L, Walters K, Petersen I. Identifying periods of acceptable computer usage in primary care research databases. Pharmacoepidemiol Drug Saf. 2013;22(November 2012):64–9.

(b) Maguire A, Blak BT, Thompson M. The importance of defining periods of complete mortality reporting for research using automated data from primary care. Vol. 18, Pharmacoepidemiology and drug safety. 2009.

Code lists

Anxiety diagnostic and symptom codes

Read code	Description
1B1Z.00	general nervous symptom nos
2258.00	o/e - anxious
E200000	anxiety state unspecified
E200400	chronic anxiety
E200500	recurrent anxiety
E200z00	anxiety state
E202100	agoraphobia with panic attacks
E202200	agoraphobia without history of panic disorder
E283.00	other acute stress reactions
E283000	acute situational disturbance
E283z00	other acute stress reaction nos
Eu41.00	[x]other anxiety disorders
Eu41000	[x]panic disorder [episodic paroxysmal anxiety]
Eu41300	[x]other mixed anxiety disorders
Eu41z00	[x]anxiety disorder, unspecified
Eu43y00	[x]other reactions to severe stress
Eu43z00	[x]reaction to severe stress, unspecified
E200.99	anxiety state
R2y2.00	[d]nervousness
Ryu5800	[x]state of emotional shock and stress, unspecified
1B1T.00	feeling stressed
E200111	panic attack
8G94.00	anxiety management training
E28z.00	acute stress reaction nos
Eu41y00	[x]other specified anxiety disorders
ZV4By00	occupation-related stress disorder
ZVu4100	[x]other physical and mental strain related to work
E200200	generalised anxiety disorder
E28..00	acute stress reaction
1BK..00	worried
E200100	panic disorder
R2y2.12	[d]nervous tension
R2y2.11	feeling nervous
E292400	adjustment reaction with anxious mood
1B1V.00	c/o - panic attack
388N.00	had scale: anxiety score
1B13.00	anxiousness
388b.00	depression anxiety stress scales anxiety score
225J.00	o/e - panic attack

38GQ.00	short health anxiety inventory
1B12.12	nervous tension
E202.11	social phobic disorders
1B12.11	nerves
1B12.00	nervousness
8HHp.00	referral for guided self-help for anxiety
R00zW00	emotional stress
Eu43011	[x]acute crisis reaction
Eu43012	[x]acute reaction to stress
Eu43000	[x]acute stress reaction
Eu40000	agoraphobia
Eu40011	[x]agoraphobia without history of panic disorder
Eu40111	[x]anthropophobia
Eu41y11	[x]anxiety hysteria
Eu41111	anxiety neurosis
Eu41z11	[x]anxiety nos
Eu41112	[x]anxiety reaction
Eu41113	[x]anxiety state
Eu43014	[x]crisis state
Eu41100	[x]generalized anxiety disorder
Eu41211	[x]mild anxiety depression
Eu41200	[x]mixed anxiety and depressive disorder
Eu05400	organic anxiety disorder
Eu41011	[x]panic attack
Eu40012	[x]panic disorder with agoraphobia
Eu41012	[x]panic state
Eu34114	[x]persistant anxiety depression
Eu93100	[x]phobic anxiety disorder of childhood
Eu43100	post-traumatic stress disorder
Eu43015	[x]psychic shock
Eu43.00	stress and adjustment reaction
Eu40112	[x]social neurosis
Eu40100	social phobia
Eu43111	[x]traumatic neurosis
E200.00	anxiety disorder
E200300	mixed anxiety and depressive disorder
1B13.11	anxiousness - symptom
1B13.12	anxious
388w.00	generalised anxiety disorder 7 item score
388w.11	gad-7 score
E202199	agoraphobia
38QN.00	gad-2 (generalised anxiety disorder 2) scale score
173f.00	anxiety about breathlessness

6897.00	anxiety screening
6897000	anxiety screening using questions
8CAZ000	patient given advice about management of anxiety
8IH8.00	gad-7 (generalized anxiety disorder 7) scale declined

Anxiety prescriptions

BNF codes: 02.04.00, 04.01.01, 04.01.02, 04.08.01

Depression symptom and diagnostic codes

Read code	Description
13Y3.00	manic-depression association member
1B17.00	depressed
1B17.11	c/o - feeling depressed
1B1U.00	symptoms of depression
1B1U.11	depressive symptoms
1BT..00	depressed mood
1BT..11	low mood
1BT..12	sad mood
1JJ..00	suspected depression
2257.00	o/e - depressed
388Z.00	depression anxiety stress scales depression score
62T1.00	puerperal depression
6891.00	depression screening
6896.00	depression screening using questions
6G00.00	postnatal depression counselling
8BK0.00	depression management programme
8CAa.00	patient given advice about management of depression
8HHq.00	referral for guided self-help for depression
8HHq000	referral for depression self-help video
8IH5200	referral for guided self-help for depression declined
9H90.00	depression annual review
9H91.00	depression medication review
9H92.00	depression interim review
9HA0.00	on depression register
9kQ..00	on full dose long term treatment depression - enh serv admin
9kQ..11	on full dose long term treatment for depression
E02y300	drug-induced depressive state
E112.00	major depression, single episode
E11..12	depressive psychoses
E112000	single major depressive episode, unspecified
E112100	mild major depression, single episode
E112.11	agitated depression

E112200	moderate major depression, single episode
E112.12	endogenous depression first episode
E112300	severe major depression, single episode
E112.13	endogenous depression first episode
E112400	single major depressive episode, severe, with psychosis
E112.14	endogenous depression
E112500	major depression single episode, in partial remission
E112600	single episode of major depression in full remission
E112z00	single major depressive episode nos
E113.00	recurrent major depressive episodes
E113000	recurrent major depressive episodes, unspecified
E113100	recurrent major depressive episodes, mild
E113.11	endogenous depression - recurrent
E113200	recurrent major depressive episodes, moderate
E113300	recurrent major depressive episodes, severe
E113400	recurrent major depressive episodes, severe, with psychosis
E113500	recurrent major depressive episodes,partial/unspec remission
E113600	recurrent major depression in full remission
E113700	recurrent depression
E113z00	recurrent major depressive episode nos
E11y200	atypical depressive disorder
E11z200	masked depression
E130.00	reactive depressive psychosis
E130.11	psychotic reactive depression
E135.00	agitated depression
E204.00	reactive depression (situational)
E204.11	postpartum depression
E204.99	reactive (neurotic) depression
E290.00	brief depressive adjustment reaction
E290z00	brief depressive reaction nos
E291.00	prolonged depressive adjustment reaction
E2B..00	depressive disorder
E2B0.00	postviral depression
E2B1.00	chronic depression
E2B..98	depression
E2B..99	depression nos
Eu32.00	depressive episode
Eu32000	mild depression
Eu32099	mild depression
Eu32100	[x]moderate depressive episode
Eu32.11	[x]single episode of depressive reaction
Eu32199	moderate depression
Eu32200	[x]severe depressive episode without psychotic symptoms

Eu32.12	[x]single episode of psychogenic depression
Eu32211	[x] single episode agitated depression without psychotic symptoms
Eu32212	severe depression
Eu32213	[x]single episode vital depression w'out psychotic symptoms
Eu32299	severe depression
Eu32300	[x]severe depressive episode with psychotic symptoms
Eu32.13	[x]single episode of reactive depression
Eu32311	[x]single episode of major depression and psychotic symptoms
Eu32312	[x]single episode of psychogenic depressive psychosis
Eu32313	[x]single episode of psychotic depression
Eu32314	[x]single episode of reactive depressive psychosis
Eu32400	[x]mild depression
Eu32500	mild major depression
Eu32600	moderate major depression
Eu32700	severe major depression without psychotic features
Eu32800	severe major depression with psychotic features
Eu32900	[x]single major depressive episode, severe, with psychosis, psychosis in remission
Eu32A00	[x]recurrent major depressive episodes, severe, with psychosis, psychosis in remission
Eu32B00	antenatal depression
Eu32y00	[x]other depressive episodes
Eu32y11	[x]atypical depression
Eu32y12	[x]single episode of masked depression nos
Eu32z00	[x]depressive episode, unspecified
Eu32z11	depression
Eu32z12	[x]depressive disorder nos
Eu32z13	[x]prolonged single episode of reactive depression
Eu32z14	reactive depression
Eu33.00	[x]recurrent depressive disorder
Eu33000	[x]recurrent depressive disorder, current episode mild
Eu33100	[x]recurrent depressive disorder, current episode moderate
Eu33.11	[x]recurrent episodes of depressive reaction
Eu33200	[x]recurr depress disorder cur epi severe without psyc sympt
Eu33.12	[x]recurrent episodes of psychogenic depression
Eu33211	[x]endogenous depression without psychotic symptoms
Eu33212	[x]major depression, recurrent without psychotic symptoms
Eu33213	[x] manic-depressive psychosis, depressed type without psychotic symptoms
Eu33214	[x]vital depression, recurrent without psychotic symptoms
Eu33300	[x]recurrent depress disorder cur epi severe with psyc symp
Eu33.13	[x]recurrent episodes of reactive depression
Eu33311	[x]endogenous depression with psychotic symptoms
Eu33313	[x]recurr severe episodes/major depression+psychotic symptom
Eu33314	[x]recurr severe episodes/psychogenic depressive psychosis
Eu33315	[x]recurrent severe episodes of psychotic depression

Eu33400	[x]recurrent depressive disorder, currently in remission
Eu33y00	[x]other recurrent depressive disorders
Eu33z00	[x]recurrent depressive disorder, unspecified
Eu33z11	[x]monopolar depression nos
Eu3y111	[x]recurrent brief depressive episodes
Eu43400	chronic post-traumatic stress disorder following military combat
Eu53011	[x]postnatal depression nos
Eu53012	[x]postpartum depression nos
Eu92000	[x]depressive conduct disorder
R007z13	[d]postoperative depression
R00zW00	emotional stress
Ryu5800	[x]state of emotional shock and stress, unspecified

Muscle spasticity

Read code	Description
F141.00	Hereditary spastic paraplegia
F221.00	Spastic hemiplegia
Q48y300	Congenital hypertonia
F23..11	Congenital spastic cerebral palsy
F230100	Cerebral palsy with spastic diplegia
F23y200	Spastic cerebral palsy
F240100	Spastic tetraplegia
F241100	Spastic paraplegia
N23yC00	Contracture of muscle

Alcohol withdrawal

Read code	Description
8BA8.00	alcohol detoxification
E01y000	Alcohol withdrawal syndrome
Eu10300	[X]Mental and behavioural disorders due to use of alcohol: withdrawal state
Eu10400	[X]Mental and behavioural disorders due to use of alcohol: withdrawal state with delirium
Z191.00	alcohol detoxification

Psychosis

Read code	Description
1464.00	H/O: schizophrenia
146D.00	H/O: manic depressive disorder
146H.00	H/O: psychosis

9H6..00	On national service framework mental health
9H8..00	On severe mental illness register
E1...00	Non-organic psychoses
E10..00	Schizophrenic disorders
E100.00	Simple schizophrenia
E100.11	Schizophrenia simplex
E100000	Unspecified schizophrenia
E100100	Subchronic schizophrenia
E100200	Chronic schizophrenic
E100300	Acute exacerbation of subchronic schizophrenia
E100400	Acute exacerbation of chronic schizophrenia
E100500	Schizophrenia in remission
E100z00	Simple schizophrenia NOS
E101.00	Hebephrenic schizophrenia
E101000	Unspecified hebephrenic schizophrenia
E101100	Subchronic hebephrenic schizophrenia
E101200	Chronic hebephrenic schizophrenia
E101300	Acute exacerbation of subchronic hebephrenic schizophrenia
E101400	Acute exacerbation of chronic hebephrenic schizophrenia
E101500	Hebephrenic schizophrenia in remission
E101z00	Hebephrenic schizophrenia NOS
E102.00	Catatonic schizophrenia
E102000	Unspecified catatonic schizophrenia
E102100	Subchronic catatonic schizophrenia
E102200	Chronic catatonic schizophrenia
E102300	Acute exacerbation of subchronic catatonic schizophrenia
E102400	Acute exacerbation of chronic catatonic schizophrenia
E102500	Catatonic schizophrenia in remission
E102z00	Catatonic schizophrenia NOS
E103.00	Paranoid schizophrenia
E103000	Unspecified paranoid schizophrenia
E103100	Subchronic paranoid schizophrenia
E103200	Chronic paranoid schizophrenia
E103300	Acute exacerbation of subchronic paranoid schizophrenia
E103400	Acute exacerbation of chronic paranoid schizophrenia
E103500	Paranoid schizophrenia in remission
E103z00	Paranoid schizophrenia NOS
E104.00	Acute schizophrenic episode
E104.11	Oneirophrenia
E105.00	Latent schizophrenia
E105000	Unspecified latent schizophrenia
E105100	Subchronic latent schizophrenia
E105200	Chronic latent schizophrenia

E105300	Acute exacerbation of subchronic latent schizophrenia
E105400	Acute exacerbation of chronic latent schizophrenia
E105500	Latent schizophrenia in remission
E105z00	Latent schizophrenia NOS
E106.00	Residual schizophrenia
E106.11	Restzustand - schizophrenia
E107.00	Schizo-affective schizophrenia
E107.11	Cyclic schizophrenia
E107000	Unspecified schizo-affective schizophrenia
E107100	Subchronic schizo-affective schizophrenia
E107200	Chronic schizo-affective schizophrenia
E107300	Acute exacerbation subchronic schizo-affective schizophrenia
E107400	Acute exacerbation of chronic schizo-affective schizophrenia
E107500	Schizo-affective schizophrenia in remission
E107z00	Schizo-affective schizophrenia NOS
E10y.00	Other schizophrenia
E10y.11	Cenesthopathic schizophrenia
E10y000	Atypical schizophrenia
E10y100	Coenesthopathic schizophrenia
E10yz00	Other schizophrenia NOS
E10z.00	Schizophrenia NOS
E11..00	Affective psychoses
E11..11	Bipolar psychoses
E11..12	Depressive psychoses
E11..13	Manic psychoses
E110.00	Manic disorder, single episode
E110.11	Hypomanic psychoses
E110000	Single manic episode, unspecified
E110100	Single manic episode, mild
E110200	Single manic episode, moderate
E110300	Single manic episode, severe without mention of psychosis
E110400	Single manic episode, severe, with psychosis
E110500	Single manic episode in partial or unspecified remission
E110600	Single manic episode in full remission
E110z00	Manic disorder, single episode NOS
E111.00	Recurrent manic episodes
E111000	Recurrent manic episodes, unspecified
E111100	Recurrent manic episodes, mild
E111200	Recurrent manic episodes, moderate
E111300	Recurrent manic episodes, severe without mention psychosis
E111400	Recurrent manic episodes, severe, with psychosis
E111500	Recurrent manic episodes, partial or unspecified remission
E111600	Recurrent manic episodes, in full remission

E111z00	Recurrent manic episode NOS
E112400	Single major depressive episode, severe, with psychosis
E113400	Recurrent major depressive episodes, severe, with psychosis
E114.00	Bipolar affective disorder, currently manic
E114.11	Manic-depressive - now manic
E114000	Bipolar affective disorder, currently manic, unspecified
E114100	Bipolar affective disorder, currently manic, mild
E114200	Bipolar affective disorder, currently manic, moderate
E114300	Bipolar affect disord, currently manic, severe, no psychosis
E114400	Bipolar affect disord, currently manic,severe with psychosis
E114500	Bipolar affect disord,currently manic, part/unspec remission
E114600	Bipolar affective disorder, currently manic, full remission
E114z00	Bipolar affective disorder, currently manic, NOS
E115.00	Bipolar affective disorder, currently depressed
E115.11	Manic-depressive - now depressed
E115000	Bipolar affective disorder, currently depressed, unspecified
E115100	Bipolar affective disorder, currently depressed, mild
E115200	Bipolar affective disorder, currently depressed, moderate
E115300	Bipolar affect disord, now depressed, severe, no psychosis
E115400	Bipolar affect disord, now depressed, severe with psychosis
E115500	Bipolar affect disord, now depressed, part/unspec remission
E115600	Bipolar affective disorder, now depressed, in full remission
E115z00	Bipolar affective disorder, currently depressed, NOS
E116000	Mixed bipolar affective disorder E116.00 Mixed bipolar affective disorder, unspecified
E116200	Mixed bipolar affective disorder, mild E116100 Mixed bipolar affective disorder, moderate
E116300	Mixed bipolar affective disorder, severe, without psychosis
E116400	Mixed bipolar affective disorder, severe, with psychosis
E116500	Mixed bipolar affective disorder, partial/unspec remission
E116600	Mixed bipolar affective disorder, in full remission
E116z00	Mixed bipolar affective disorder, NOS
E117.00	Unspecified bipolar affective disorder
E117000	Unspecified bipolar affective disorder, unspecified
E117100	Unspecified bipolar affective disorder, mild
E117200	Unspecified bipolar affective disorder, moderate
E117300	Unspecified bipolar affective disorder, severe, no psychosis
E117400	Unspecified bipolar affective disorder,severe with psychosis
E117500	Unspecified bipolar affect disord, partial/unspec remission
E117600	Unspecified bipolar affective disorder, in full remission
E117z00	Unspecified bipolar affective disorder, NOS
E11y.00	Other and unspecified manic-depressive psychoses
E11y000	Unspecified manic-depressive psychoses
E11y100	Atypical manic disorder

E11y300	Other mixed manic-depressive psychoses
E11yz00	Other and unspecified manic-depressive psychoses NOS
E11z.00	Other and unspecified affective psychoses
E11z000	Unspecified affective psychoses NOS
E11zz00	Other affective psychosis NOS
E12..00	Paranoid states
E120.00	Simple paranoid state
E121.00	Chronic paranoid psychosis
E121.11	Sander's disease
E122.00	Paraphrenia
E123.00	Shared paranoid disorder
E123.11	Folie a deux
E12y.00	Other paranoid states
E12y000	Paranoia querulans
E12yz00	Other paranoid states NOS
E12z.00	Paranoid psychosis NOS
E13..00	Other nonorganic psychoses
E13..11	Reactive psychoses
E130.00	Reactive depressive psychosis
E130.11	Psychotic reactive depression
E131.00	Acute hysterical psychosis
E133.00	Acute paranoid reaction
E133.11	Bouffee delirante
E134.00	Psychogenic paranoid psychosis
E13y.00	Other reactive psychoses
E13y000	Psychogenic stupor
E13y100	Brief reactive psychosis
E13yz00	Other reactive psychoses NOS
E13z.00	Nonorganic psychosis NOS
E13z.11	Psychotic episode NOS
E1y..00	Other specified non-organic psychoses
E1z..00	Non-organic psychosis NOS
E212200	Schizotypal personality
Eu2..00	[X]Schizophrenia, schizotypal and delusional disorders
Eu20000	[X]Schizophrenia Eu20.00 [X]Paranoid schizophrenia
Eu20011	[X]Paraphrenic schizophrenia
Eu20100	[X]Hebephrenic schizophrenia
Eu20111	[X]Disorganised schizophrenia
Eu20200	[X]Catatonic schizophrenia
Eu20211	[X]Catatonic stupor
Eu20212	[X]Schizophrenic catalepsy
Eu20213	[X]Schizophrenic catatonia
Eu20214	[X]Schizophrenic flexibilatis cerea

Eu20300	[X]Undifferentiated schizophrenia
Eu20311	[X]Atypical schizophrenia
Eu20400	[X]Post-schizophrenic depression
Eu20500	[X]Residual schizophrenia
Eu20511	[X]Chronic undifferentiated schizophrenia
Eu20512	[X]Restzustand schizophrenic
Eu20600	[X]Simple schizophrenia
Eu20y00	[X]Other schizophrenia
Eu20y11	[X]Cenesthopathic schizophrenia
Eu20y12	[X]Schizophreniform disord NOS
Eu20y13	[X]Schizophrenifrm psychos NOS
Eu20z00	[X]Schizophrenia, unspecified
Eu21.00	[X]Schizotypal disorder
Eu21.11	[X]Latent schizophrenic reaction
Eu21.12	[X]Borderline schizophrenia
Eu21.13	[X]Latent schizophrenia
Eu21.14	[X]Prepsychotic schizophrenia
Eu21.15	[X]Prodromal schizophrenia
Eu21.16	[X]Pseudoneurotic schizophrenia
Eu21.17	[X]Pseudopsychopathic schizophrenia
Eu21.18	[X]Schizotypal personality disorder
Eu22.00	[X]Persistent delusional disorders
Eu22000	[X]Delusional disorder
Eu22011	[X]Paranoid psychosis
Eu22012	[X]Paranoid state
Eu22013	[X]Paraphrenia - late
Eu22014	[X]Sensitiver Beziehungswahn
Eu22015	[X]Paranoia
Eu22100	[X]Delusional misidentification syndrome
Eu22111	[X]Capgras syndrome
Eu22200	[X]Cotard syndrome
Eu22y00	[X]Other persistent delusional disorders
Eu22y11	[X]Delusional dysmorphophobia
Eu22y12	[X]Involutional paranoid state
Eu22y13	[X]Paranoia querulans
Eu22z00	[X]Persistent delusional disorder, unspecified
Eu23.00	[X]Acute and transient psychotic disorders
Eu23000	[X]Acute polymorphic psychot disord without symp of schizoph
Eu23011	[X]Bouffee delirante
Eu23012	[X]Cycloid psychosis
Eu23100	[X]Acute polymorphic psychot disord with symp of schizophren
Eu23111	[X]Bouffee delirante with symptoms of schizophrenia
Eu23112	[X]Cycloid psychosis with symptoms of schizophrenia

Eu23200	[X]Acute schizophrenia-like psychotic disorder
Eu23211	[X]Brief schizophreniform disorder
Eu23212	[X]Brief schizophrenifrm psych
Eu23213	[X]Oneirophrenia
Eu23214	[X]Schizophrenic reaction
Eu23300	[X]Other acute predominantly delusional psychotic disorders
Eu23312	[X]Psychogenic paranoid psychosis
Eu23y00	[X]Other acute and transient psychotic disorders
Eu23z00	[X]Acute and transient psychotic disorder, unspecified
Eu23z11	[X]Brief reactive psychosis NOS
Eu23z12	[X]Reactive psychosis
Eu24.00	[X]Induced delusional disorder
Eu24.11	[X]Folie a deux
Eu24.12	[X]Induced paranoid disorder
Eu24.13	[X]Induced psychotic disorder
Eu25.00	[X]Schizoaffective disorders
Eu25000	[X]Schizoaffective disorder, manic type
Eu25011	[X]Schizoaffective psychosis, manic type
Eu25012	[X]Schizophreniform psychosis, manic type
Eu25100	[X]Schizoaffective disorder, depressive type
Eu25111	[X]Schizoaffective psychosis, depressive type
Eu25112	[X]Schizophreniform psychosis, depressive type
Eu25200	[X]Schizoaffective disorder, mixed type
Eu25211	[X]Cyclic schizophrenia
Eu25212	[X]Mixed schizophrenic and affective psychosis
Eu25y00	[X]Other schizoaffective disorders
Eu25z00	[X]Schizoaffective disorder, unspecified
Eu25z11	[X]Schizoaffective psychosis NOS
Eu2y.00	[X]Other nonorganic psychotic disorders
Eu2y.11	[X]Chronic hallucinatory psychosis
Eu2z.00	[X]Unspecified nonorganic psychosis
Eu2z.11	[X]Psychosis NOS
Eu30.00	[X]Manic episode
Eu30.11	[X]Bipolar disorder, single manic episode
Eu30000	[X]Hypomania
Eu30100	[X]Mania without psychotic symptoms
Eu30200	[X]Mania with psychotic symptoms
Eu30211	[X]Mania with mood-congruent psychotic symptoms
Eu30212	[X]Mania with mood-incongruent psychotic symptoms
Eu30y00	[X]Manic stupor Eu30213 [X]Other manic episodes
Eu30z00	[X]Manic episode, unspecified
Eu30z11	[X]Mania NOS
Eu31.00	[X]Bipolar affective disorder

Eu31.11	[X]Manic-depressive illness
Eu31.12	[X]Manic-depressive psychosis
Eu31.13	[X]Mainc-depressive reaction
Eu31000	[X]Bipolar affective disorder, current episode hypomanic
Eu31100	[X]Bipolar affect disorder cur epi manic wout psychotic symp
Eu31200	[X]Bipolar affect disorder cur epi manic with psychotic symp
Eu31300	[X]Bipolar affect disorder cur epi mild or moderate depressn
Eu31400	[X]Bipol aff disord, curr epis sev depress, no psychot symp
Eu31500	[X]Bipolar affect dis cur epi severe depres with psyc symp
Eu31600	[X]Bipolar affective disorder, current episode mixed
Eu31700	[X]Bipolar affective disorder, currently in remission
Eu31y00	[X]Other bipolar affective disorders
Eu31y11	[X]Bipolar II disorder
Eu31y12	[X]Recurrent manic episodes
Eu31z00	[X]Bipolar affective disorder, unspecified
Eu32300	[X]Severe depressive episode with psychotic symptoms
Eu32311	[X]Single episode of major depression and psychotic symptoms
Eu32312	[X]Single episode of psychogenic depressive psychosis
Eu32313	[X]Single episode of psychotic depression
Eu32314	[X]Single episode of reactive depressive psychosis
Eu32800	[X]Major depression, severe with psychotic symptoms
Eu33213	[X]Manic-depress psychosis,depressd,no psychotic symptoms
Eu33300	[X]Recurrent depress disorder cur epi severe with psyc symp
Eu33311	[X]Endogenous depression with psychotic symptoms
Eu33312	[X]Manic-depress psychosis,depressed type+psychotic symptoms
Eu33313	[X]Recurr severe episodes/major depression+psychotic symptom
Eu33314	[X]Recurr severe episodes/psychogenic depressive psychosis
Eu33315	[X]Recurrent severe episodes of psychotic depression
Eu33316	[X]Recurrent severe episodes/reactive depressive psychosis
Eu3z.11	[X]Affective psychosis NOS
Eu44.14	[X]Hysterical psychosis
Eu84314	[X]Symbiotic psychosis
ZS7C611	Profile of mood states, bipolar ZRby100 Schizophrenic language
ZV11000	[V]Personal history of schizophrenia
ZV11111	[V]Personal history of manic-depressive psy
ZV11112	[V]Personal history of manic-depressive psy

High blood pressure

**Read code**	**Description**
662..12	Hypertension monitoring
6627.00	Good hypertension control
6628.00	Poor hypertension control

662b.00	Moderate hypertension control
662c.00	Hypertension six month review
662d.00	Hypertension annual review
662F.00	Hypertension treatm. started
662G.00	Hypertensive treatm.changed
662O.00	On treatment for hypertension
662P.00	Hypertension monitoring
8B26.00	Antihypertensive therapy
8BL0.00	Patient on maximal tolerated antihypertensive therapy
8CR4.00	Hypertension clinical management plan
8HT5.00	Referral to hypertension clinic
9N03.00	Seen in hypertension clinic
9N1y200	Seen in hypertension clinic
9OI1.00	Attends hypertension monitor.
9OIA.00	Hypertension monitor.chck done
9OIA.11	Hypertension monitored
F421300	Hypertensive retinopathy
G2...00	Hypertensive disease
G20..00	Essential hypertension
G200.00	Malignant essential hypertension
G201.00	Benign essential hypertension
G20..11	High blood pressure
G202.00	Systolic hypertension
G20z.00	Essential hypertension NOS
G20z.11	Hypertension NOS
G2...11	BP - hypertensive disease
G24..00	Secondary hypertension
G240.00	Secondary malignant hypertension
G240000	Secondary malignant renovascular hypertension
G240z00	Secondary malignant hypertension NOS
G241.00	Secondary benign hypertension
G241000	Secondary benign renovascular hypertension
G241z00	Secondary benign hypertension NOS
G244.00	Hypertension secondary to endocrine disorders
G24z.00	Secondary hypertension NOS
G24z000	Secondary renovascular hypertension NOS
G24zz00	Secondary hypertension NOS
G2y..00	Other specified hypertensive disease
G2z..00	Hypertensive disease NOS
G672.11	Hypertensive crisis
Gyu2000	[X]Other secondary hypertension
Gyu2100	[X]Hypertension secondary to other renal disorders

Irregular heartbeat

Read code	Description
14AN.00	h/o: atrial fibrillation
14AR.00	history of atrial flutter
212R.00	atrial fibrillation resolved
327..00	ecg: supraventricular arrhythmia
3272.00	ecg: atrial fibrillation
3273.00	ecg: atrial flutter
662S.00	atrial fibrillation monitoring
6A9..00	atrial fibrillation annual review
8CMW200	atrial fibrillation care pathway
8HTy.00	referral to atrial fibrillation clinic
9hF..00	exception reporting: atrial fibrillation quality indicators
9hF1.00	excepted from atrial fibrillation qual indic: inform dissent
9Os..00	atrial fibrillation monitoring administration
9Os0.00	atrial fibrillation monitoring first letter
9Os1.00	atrial fibrillation monitoring second letter
9Os2.00	atrial fibrillation monitoring third letter
9Os3.00	atrial fibrillation monitoring verbal invite
9Os4.00	atrial fibrillation monitoring telephone invite
G55A.11	tachycardia-induced cardiomyopathy
G56..00	conduction disorders
G56..11	conduction disorders of heart
G56y.00	other conduction disorders
G56zz00	conduction disorders nos
G57..00	cardiac dysrhythmias
G57..11	cardiac arrhythmias
G573.00	atrial fibrillation and flutter
G573000	atrial fibrillation
G573300	non-rheumatic atrial fibrillation
G573400	permanent atrial fibrillation
G573500	persistent atrial fibrillation
G573z00	atrial fibrillation and flutter nos
G57y.00	other cardiac dysrhythmias
G57y900	supraventricular tachycardia nos
G57yz00	other cardiac dysrhythmia nos
G57z.00	cardiac dysrhythmia nos

Heart failure

Read code	Description
14A6.00	h/o: heart failure

14AM.00	h/o: heart failure in last year
1736.00	paroxysmal nocturnal dyspnoea
1J60.00	suspected heart failure
1O1..00	heart failure confirmed
388D.00	new york heart assoc classification heart failure symptoms
585f.00	echocardiogram shows left ventricular systolic dysfunction
585g.00	echocardiogram shows left ventricular diastolic dysfunction
661M500	heart failure self-management plan agreed
662f.00	new york heart association classification - class i
662g.00	new york heart association classification - class ii
662h.00	new york heart association classification - class iii
662i.00	new york heart association classification - class iv
662p.00	heart failure 6 month review
662T.00	congestive heart failure monitoring
662W.00	heart failure annual review
679W100	education about deteriorating heart failure
679X.00	heart failure education
67D4.00	heart failure information given to patient
8B29.00	cardiac failure therapy
8CeC.00	preferred place of care for next exacerbation heart failure
8CL3.00	heart failure care plan discussed with patient
8CMK.00	has heart failure management plan
8CMW800	heart failure clinical pathway
8H2S.00	admit heart failure emergency
8HBE.00	heart failure follow-up
8Hg8.00	discharge from practice nurse heart failure clinic
8HgD.00	discharge from heart failure nurse service
8HHb.00	referral to heart failure nurse
8HHz.00	referral to heart failure exercise programme
8Hk0.00	referred to heart failure education group
8HTL.00	referral to heart failure clinic
8HTL000	referral to rapid access heart failure clinic
8IE0.00	referral to heart failure education group declined
8IE1.00	referral to heart failure exercise programme declined
9h1..00	exception reporting: lvd quality indicators
9h11.00	excepted from lvd quality indicators: patient unsuitable
9h12.00	excepted from lvd quality indicators: informed dissent
9hH..00	exception reporting: heart failure quality indicators
9hH0.00	excepted heart failure quality indicators: patient unsuitabl
9hH1.00	excepted heart failure quality indicators: informed dissent
9N0k.00	seen in heart failure clinic
9N2p.00	seen by community heart failure nurse
9N4s.00	did not attend practice nurse heart failure clinic

9N4w.00	did not attend heart failure clinic
9N6T.00	referred by heart failure nurse specialist
9On..00	left ventricular dysfunction monitoring administration
9On0.00	left ventricular dysfunction monitoring first letter
9On1.00	left ventricular dysfunction monitoring second letter
9On2.00	left ventricular dysfunction monitoring third letter
9On3.00	left ventricular dysfunction monitoring verbal invite
9On4.00	left ventricular dysfunction monitoring telephone invite
9Or..00	heart failure monitoring administration
9Or0.00	heart failure review completed
9Or1.00	heart failure monitoring telephone invite
9Or2.00	heart failure monitoring verbal invite
9Or3.00	heart failure monitoring first letter
9Or4.00	heart failure monitoring second letter
9Or5.00	heart failure monitoring third letter
G1yz100	rheumatic left ventricular failure
G210.00	malignant hypertensive heart disease
G210100	malignant hypertensive heart disease with ccf
G211100	benign hypertensive heart disease with ccf
G21z100	hypertensive heart disease nos with ccf
G230.00	malignant hypertensive heart and renal disease
G232.00	hypertensive heart&renal dis wth (congestive) heart failure
G234.00	hyperten heart&renal dis+both(congestv)heart and renal fail
G400.00	acute cor pulmonale
G41z.11	chronic cor pulmonale
G554000	congestive cardiomyopathy
G554011	congestive obstructive cardiomyopathy
G557100	beriberi heart disease
G58..00	heart failure
G580.00	congestive heart failure
G580000	acute congestive heart failure
G580100	chronic congestive heart failure
G580.11	congestive cardiac failure
G580.12	right heart failure
G580.13	right ventricular failure
G580.14	biventricular failure
G580200	decompensated cardiac failure
G580300	compensated cardiac failure
G580400	congestive heart failure due to valvular disease
G581.00	left ventricular failure
G581000	acute left ventricular failure
G58..11	cardiac failure
G581.11	asthma - cardiac

G581.13	impaired left ventricular function
G582.00	acute heart failure
G583.00	heart failure with normal ejection fraction
G583.11	hfnef - heart failure with normal ejection fraction
G583.12	heart failure with preserved ejection fraction
G584.00	right ventricular failure
G58z.00	heart failure nos
G58z.11	weak heart
G58z.12	cardiac failure nos
G5y4z00	post cardiac operation heart failure nos
G5yy900	left ventricular systolic dysfunction
G5yyA00	left ventricular diastolic dysfunction
G5yyB00	right ventricular diastolic dysfunction
Q48y100	congenital cardiac failure
R2y1000	[d]cardiorespiratory failure
SP11111	heart failure as a complication of care
ZRad.00	new york heart assoc classification heart failure symptoms

Heart attack

**Read code**	**Description**
323..00	ECG: myocardial infarction
323Z.00	ECG: myocardial infarct NOS
G30..00	Acute myocardial infarction
G300.00	Acute anterolateral infarction
G301.00	Other specified anterior myocardial infarction
G301000	Acute anteroapical infarction
G301100	Acute anteroseptal infarction
G30..12	Coronary thrombosis
G30..13	Cardiac rupture following myocardial infarction (MI)
G30..15	MI - acute myocardial infarction
G30..16	Thrombosis - coronary
G30..17	Silent myocardial infarction
G301z00	Anterior myocardial infarction NOS
G302.00	Acute inferolateral infarction
G303.00	Acute inferoposterior infarction
G304.00	Posterior myocardial infarction NOS
G305.00	Lateral myocardial infarction NOS
G306.00	True posterior myocardial infarction
G307.00	Acute subendocardial infarction
G307000	Acute non-Q wave infarction
G307100	Acute non-ST segment elevation myocardial infarction
G308.00	Inferior myocardial infarction NOS

G309.00	Acute Q-wave infarct
G30B.00	Acute posterolateral myocardial infarction
G30X.00	Acute transmural myocardial infarction of unspecif site
G30X000	Acute ST segment elevation myocardial infarction
G30y.00	Other acute myocardial infarction
G30y100	Acute papillary muscle infarction
G30y200	Acute septal infarction
G30yz00	Other acute myocardial infarction NOS
G30z.00	Acute myocardial infarction NOS
G311000	Myocardial infarction aborted
G311011	MI - myocardial infarction aborted
G311500	Acute coronary syndrome
G35..00	Subsequent myocardial infarction
G350.00	Subsequent myocardial infarction of anterior wall
G351.00	Subsequent myocardial infarction of inferior wall
G353.00	Subsequent myocardial infarction of other sites
G35X.00	Subsequent myocardial infarction of unspecified site
G360.00	Haemopericardium/current comp folow acut myocard infarct
G362.00	Ventric septal defect/curr comp fol acut myocardal infarctn
G363.00	Ruptur cardiac wall w'out haemopericard/cur comp fol ac MI
G364.00	Ruptur chordae tendinae/curr comp fol acute myocard infarct
G365.00	Rupture papillary muscle/curr comp fol acute myocard infarct
G38..00	Postoperative myocardial infarction
G380.00	Postoperative transmural myocardial infarction anterior wall
G381.00	Postoperative transmural myocardial infarction inferior wall
G384.00	Postoperative subendocardial myocardial infarction
G38z.00	Postoperative myocardial infarction; unspecified
G574011	Cardiac arrest-ventricular fibrillation
G575.00	Cardiac arrest
G575000	Cardiac arrest with successful resuscitation
G575100	Sudden cardiac death; so described
G575.11	Cardio-respiratory arrest
G575.12	Asystole
G575z00	Cardiac arrest; unspecified
Gyu3400	[X]Acute transmural myocardial infarction of unspecif site
Gyu3600	[X]Subsequent myocardial infarction of unspecified site

Thyrotoxicosis

**Read code**	**Description**
C02..00	Hyperthyroidism / thyrotoxicosis
C024.00	Thyrotoxicosis from ectopic thyroid nodule
C024000	Thyrotoxicosis from ectopic thyroid nodule with no crisis

C024100	Thyrotoxicosis from ectopic thyroid nodule with crisis
C024z00	Thyrotoxicosis from ectopic thyroid nodule NOS
C02y.00	Thyrotoxicosis of other specified origin
C02y000	Thyrotoxicosis of other specified origin with no crisis
C02y100	Thyrotoxicosis of other specified origin with crisis
C02y200	Thyrotoxicosis factitia
C02y300	Thyroid crisis
C02yz00	Thyrotoxicosis of other specified origin NOS
C02z.00	Thyrotoxicosis without mention of goitre or other cause
C02z000	Thyrotoxicosis without mention of goitre or cause no crisis
C02z100	Thyrotoxicosis without mention of goitre, cause with crisis
C02zz00	Thyrotoxicosis NOS
C05y400	Chronic thyroiditis with transient thyrotoxicosis
Cyu1300	[X]Other thyrotoxicosis
F381600	Myasthenic syndrome due to thyrotoxicosis
F395400	Myopathy due to thyrotoxicosis
F4G2000	Thyrotoxic exophthalmos
G557500	Thyrotoxic heart disease

Migraine

**Read code**	**Description**
1474.00	H/O: migraine
1474000	H/O migraine with aura
1967.00	Abdominal migraine - symptom
8B6N.00	Migraine prophylaxis
F26..00	Migraine
F260.00	Classical migraine
F260.11	Migraine with aura
F261.00	Common migraine
F261000	Atypical migraine
F261.11	Migraine without aura
F261z00	Common migraine NOS
F262.00	Migraine variants
F262200	Abdominal migraine
F262300	Basilar migraine
F262400	Ophthalmic migraine
F262500	Periodic migrainous neuralgia
F262800	Migraine induced by oestrogen contraceptive
F262z00	Migraine variant NOS
F26y.00	Other forms of migraine
F26y000	Hemiplegic migraine
F26y100	Ophthalmoplegic migraine

F26y111	Moebius' ophthalmoplegic migraine
F26y200	Status migrainosus
F26y300	Complicated migraine
F26yz00	Other forms of migraine NOS
F26z.00	Migraine NOS
Fyu5300	[X]Other migraine
K584.11	Migraine - menstrual
R090D00	[D]Abdominal migraine

Tremor

**Read code**	**Description**
R010300	[D]Tremor NOS
F131100	Familial tremor
F131000	Benign essential tremor
R20..11	[D]Senile tremor
2976.00	O/E - coarse tremor - flapping
1B22.11	Tremor symptom
F131.00	Essential and other specified forms of tremor
2976.11	O/E - coarse tremor
2975.00	O/E - fine tremor
297A.00	O/E - Parkinsonian tremor
2977.00	O/E - intention tremor
E201300	Hysterical tremor
2976.12	O/E - flapping tremor
1B22.00	Has a tremor
F131z00	Essential and other specified forms of tremor NOS
297B.00	O/E - tremor outstretched hands
297C.00	O/E - tremor of tongue
Fyu2500	[X]Other specified forms of tremor
F1y0.00	Fragile X associated tremor ataxia syndrome
Xa89r	Thyrotoxic tremor

Epilepsy

**Read code**	**Description**
1473.00	H/O: epilepsy
1B1W.00	Transient epileptic amnesia
1O30.00	Epilepsy confirmed
2823.00	O/E - petit mal fit
2824.00	O/E - focal (Jacksonian) fit
2824.11	O/E - Jacksonian fit
2825.00	O/E - psychomotor fit
2828.00	Absence seizure

667B.00	Nocturnal epilepsy
667N.00	Epilepsy severity
Eu05212	[X]Schizophrenia-like psychosis in epilepsy
Eu05y11	[X]Epileptic psychosis NOS
Eu06013	[X]Limbic epilepsy personality
Eu80300	[X]Acquired aphasia with epilepsy [Landau - Kleffner]
F132100	Progressive myoclonic epilepsy
F132200	Myoclonic encephalopathy
F25..00	Epilepsy
F250.00	Generalised nonconvulsive epilepsy
F250000	Petit mal (minor) epilepsy
F250011	Epileptic absences
F250100	pykno-epilepsy
F250200	Epileptic seizures - atonic
F250300	Epileptic seizures - akinetic
F250400	Juvenile absence epilepsy
F250500	Lennox-Gastaut syndrome
F250y00	Other specified generalised nonconvulsive epilepsy
F250z00	Generalised nonconvulsive epilepsy NOS
F251.00	Generalised convulsive epilepsy
F251000	Grand mal (major) epilepsy
F251011	Tonic-clonic epilepsy
F251100	Neonatal myoclonic epilepsy
F251111	Otohara syndrome
F251200	Epileptic seizures - clonic
F251300	Epileptic seizures - myoclonic
F251400	Epileptic seizures - tonic
F251500	Tonic-clonic epilepsy
F251600	Grand mal seizure
F251y00	Other specified generalised convulsive epilepsy
F251z00	Generalised convulsive epilepsy NOS
F252.00	Petit mal status
F253.00	Grand mal status
F253.11	Status epilepticus
F254.00	Partial epilepsy with impairment of consciousness
F254000	Temporal lobe epilepsy
F254100	Psychomotor epilepsy
F254200	Psychosensory epilepsy
F254300	Limbic system epilepsy
F254400	Epileptic automatism
F254500	Complex partial epileptic seizure
F254z00	Partial epilepsy with impairment of consciousness NOS
F255.00	Partial epilepsy without impairment of consciousness

F255000	Jacksonian; focal or motor epilepsy
F255011	Focal epilepsy
F255012	Motor epilepsy
F255100	Sensory induced epilepsy
F255200	Somatosensory epilepsy
F255300	Visceral reflex epilepsy
F255311	Partial epilepsy with autonomic symptoms
F255400	Visual reflex epilepsy
F255500	Unilateral epilepsy
F255600	Simple partial epileptic seizure
F255y00	Partial epilepsy without impairment of consciousness OS
F255z00	Partial epilepsy without impairment of consciousness NOS
F256.00	Infantile Spasms
F256000	Hypsarrhthymia
F256100	Salaam attacks
F256.11	Lightning spasms
F256.12	West Syndrome
F256z00	Infantile Spasms NOS
F257.00	Kojevnikov's epilepsy
F259.00	Early infant epileptic encephalopathy with suppression bursts
F259.11	Ohtahara syndrome
F25A.00	Juvenile myoclonic epilepsy
F25B.00	Alcohol-induced epilepsy
F25C.00	Drug-induced epilepsy
F25D.00	Menstrual epilepsy
F25E.00	Stress-induced epilepsy
F25F.00	Photosensitive epilepsy
F25G.00	Severe myoclonic epilepsy in infancy
F25X.00	Status epilepticus; unspecified
F25y.00	Other forms of epilepsy
F25y000	Cursive (running) epilepsy
F25y100	Gelastic epilepsy
F25y200	Locl-rlt(foc)(part)idiop epilep&epilptic syn seiz locl onset
F25y300	Complex partial status epilepticus
F25y400	Benign Rolandic epilepsy
F25y500	Panayiotopoulos syndrome
F25yz00	Other forms of epilepsy NOS
F25z.00	Epilepsy NOS
F25z.11	Fit (in known epileptic) NOS
Fyu5000	[X]Other generalized epilepsy and epileptic syndromes
Fyu5100	[X]Other epilepsy
Fyu5200	[X]Other status epilepticus
Fyu5900	[X]Status epilepticus; unspecified

SC20000	Traumatic epilepsy
ZS82.00	Acquired epileptic aphasia
ZS82.11	Landau-Kleffner syndrom

6-8 week infant check

Any of the following codes or an AHD code for a postnatal check identified in a child's record between weeks 5 to 15 after their date of birth to coincide with any general examination at the time of the 6-8 week check.

Read Code	Description
6421.00	Bottle fed at 6 weeks
6422.00	Breast fed at 6 weeks
6423.00	Breast fed + supp. at 6 weeks
6431.00	Bottle fed at 3 months
6432.00	Breast fed at 3 months
6433.00	Breast + supp.fed at 3 months
6471.00	Child height = 50th centile
6472.00	Child exam.: trunk/limbs NOS
6473.00	8 week exam.abnormal -for obs.
6474.00	6 week exam.abnormal -referred
6475.00	Child weight=50th-74th centile
6476.00	Child weight=75th-89th centile
6477.00	Child weight=90th-96th centile
6478.00	Barlow test
6481.00	Child HC = 50th centile
6483.00	Child exam.: squint
6484.00	Child height=25th-49th centile
6485.00	Infant feeding method
6486.00	Child height 26th - 49th centile
6487.00	Child weight 51st - 74th centile
6488.00	Child height 10th - 24th centile
6495.00	Child exam.: limbs
64...00	Child health care
64...11	8 week exam.abn.-on treatment
64...12	Child development checks
64...13	Child development examinations
64...14	Child height=50th-74th centile
64...15	Paediatric surveillance
64...16	Paediatric surveillance checks
642..00	Annual health review
647..00	Child weight centiles
647A.00	Breast/other feeding,diff.ages

647E.00	Breast fed at 4 months
647F.00	Child 8 week exam. not wanted
647G.00	8 week exam.abnormal -referred
647H.00	Child height=90th-96th centile
647I.00	Child 1 year examination
647J.00	Child weight 26th - 49th centile
647K.00	Child HC centiles NOS
647L.00	Child exam.: motor
647M.00	Child 3 month examination
647O.00	Child HC = 50th-74th centile
647Z.00	Child weight centiles NOS
648..00	Child height centiles
648..11	6 week exam.abn.-on treatment
648D.00	Child exam.: fine motor devel.
648E.00	Child height < 3rd centile
648F.00	Child height = 0.4th centile
648G.00	Child height=75th=89th centile
648H.00	Child weight < 3rd centile
648I.00	Child height 51st - 74th centile
648J.00	Child height = 25th centile
648K.00	Child exam.: language develop.
648L.00	Child height = 91st centile
648M.00	Child height 92nd - 97th centile
648Q.00	Child height = 75th centile
648Z.00	Child height centiles NOS
649..00	Child head circ. centiles
649I.00	Child height > 97th centile
649Z.00	Child height=10th-24th centile
64a..00	Child 8 week exam
64A..00	Baby length centiles
64A1.00	First smiled
64A2.00	Child weight = 25th centile
64a2.00	Mental health review
64a4.00	Child 8 week exam. normal
64A4.00	Child not examined at birth
64a5.00	Child weight=10th-24th centile
64a6.00	First tooth
64a7.00	Motor develop.- child
64a8.00	Child weight > 97th centile
64AZ.00	Infant milestones NOS
64B..00	Child exam. - birth
64B1.00	Child weight 10th - 24th centile
64B2.00	Child 6 week exam.not attended

64BZ.00	Child exam. - birth NOS
64D..00	Child 6 week exam.
64D3.00	Child birth exam. - normal
64D4.00	Child 6 week exam. normal
64D5.00	6 week exam.abnormal -for obs.
64D6.00	Child weight=25th-49th centile
64D7.00	Child exam.: bowel control
64D8.00	Child 6 to 8 week examination
64DZ.00	Child 6 week exam. NOS
64f0.00	Not smiling by 8 weeks old
64L..00	Child exam.: general/head
64L..11	Child exam.: head
64L1.00	Child exam.: general behaviour
64L2.00	Child exam.: appearance
64L3.00	Child exam.: skin
64L4.00	Child exam.: fontanelle
64L5.00	Child exam.: palate
64LZ.00	Child exam.: general/head NOS
64M1.00	Child exam.: vision
64M2.00	Child exam.: eyes
64M3.00	Child weight 76th - 90th centile
64M4.00	Child exam.: ears
64M5.00	Child exam.: hearing
64M6.00	Child exam.: speech
64N..00	Child exam.: trunk/limbs
64N..11	Child height 76th - 90th centile
64N1.00	Child exam.: heart
64N2.00	Child exam.: femoral arteries
64N3.00	Child exam.: hips
64N3000	Child weight = 0.4th centile
64N3100	Child height = 9th centile
64N4.00	Child exam.: spine
64N5.00	Child exam.: feet
64N6.00	Child exam.: herniae
64N7.00	Child exam.: testes
64N8.00	Child exam.: genitalia
64NZ.00	Child weight=3rd-9th centile
64O..11	Child weight = 50th centile
64O1.00	Child exam.:gross motor devel.
64O3.00	Child exam.: motor tone
64P..00	Child exam.: development
64P..11	First crawled
64P1.00	Infant milestones

64P2.00	Child in care statutory review meeting
64P3.00	Child exam.:social development
64P4.00	Child weight 9th centile
64PZ.00	Child exam.: development NOS
64T..00	Infant feeding at 6 weeks
64T1.00	Child weight = 91st centile
64TZ.00	Child weight = 98th centile
64U..00	Child weight = 75th centile
64Z..00	Child health care NOS
6A50.00	Other child examinations NOS
6A6..00	Ortolani's test
6AD1.00	Wound repair review
6AE..00	Multidisciplinary review
6AK..00	Other child examinations NEC

Sensitivity analyses

Results from sensitivity analysis conducting key analysis using a more sensitive definition of PNA which includes any perinatal SSRI prescription.

Table A: Maternal and infant characteristics comparing those with perinatal anxiety, antenatal anxiety and postnatal anxiety for sensitive definition of perinatal anxiety

Characteristics	Total N (% down)	Perinatal anxiety N (% across)	Antenatal anxiety N (% across)	Postnatal anxiety N (% across)
Overall	248,618	21,385 (8.6)	8,139 (3.3)	17,970 (7.2)
Maternal age (years)				
15-19	8,885 (3.6)	1,474 (16.6)	325 (3.7)	1,341 (12.1)
20-24	31,069 (12.5)	4,080 (13.1)	1,384 (4.5)	3,439 (11.1)
25-29	56,438 (22.7)	4,849 (8.6)	1,868 (3.3)	4,081 (7.2)
30-34	79,810 (32.1)	5,540 (6.9)	2,220 (2.8)	4,637 (5.8)
35-39	56,757 (22.8)	4,044 (7.1)	1,705 (3.0)	3,334 (5.9)
40-44	14,809 (6.0)	1,321 (8.9)	599 (4.0)	1,078 (7.3)
45-49	850 (0.3)	77 (9.1)	38 (4.5)	60 (7.1)
Townsend Score quintile				
1-least deprived	49,205 (23.4)	3,155 (6.4)	1,151 (2.3)	2,630 (5.3)
2	42,271 (20.1)	3,146 (7.4)	1,194 (2.8)	2,623 (6.2)
3	45,729 (21.8)	4,015 (8.8)	1,524 (3.3)	3,361 (7.4)
4	42,101 (20.0)	4,150 (9.9)	1,603 (3.8)	3,479 (8.3)
5-most deprived	30,970 (14.7)	3,400 (11.0)	1,328 (4.3)	2,870 (9.3)
Unknown	38,342	3,519 (9.2)	1,339 (3.5)	3,007 (7.8)
History of anxiety				
Recent	14,712 (5.9)	8,384 (57.0)	5,270 (35.8)	6,855 (46.6)
Previous	6,461 (2.6)	1,994 (30.9)	523 (8.1)	1,697 (26.3)
None	227,445 (91.5)	11,007 (4.8)	2,346 (1.0)	9,418 (4.1)
History of Depression				
Recent	12,284 (4.9)	7,411 (60.3)	4,826 (39.3)	6,074 (49.5)
Previous	5,085 (2.1)	1,749 (34.4)	475 (9.3)	1,520 (29.9)
None	231,249 (93.0)	12,225 (5.3)	2,838 (1.2)	10,376 (4.5)
Mode of delivery				
Vaginal delivery	75,096 (63.9)	5,545 (7.4)	2,107 (2.8)	4,621 (6.2)
Instrumental	15,586 (13.3)	1,750 (11.2)	648 (4.2)	1,487 (9.5)
Caesarean	26,821 (22.8)	2,503 (9.3)	1,013 (3.8)	2,085 (7.8)
Unknown	131,115	11,587 (8.8)	4,371 (3.3)	9,777 (7.5)
Year group				
1998-2001*	25,345 (10.2)	1,643 (6.5)	508 (2.0)	1,411 (5.6)
2002-2004	39,098 (15.7)	2,991 (7.7)	1,015 (2.6)	2,493 (6.4)
2005-2007	47,741 (19.2)	3,804 (8.0)	1,268 (2.7)	3,277 (6.8)
2008-2010	51,713 (20.8)	4,508 (8.7)	1,721 (3.3)	3,776 (7.3)
2011-2013	50,075 (20.1)	4,638 (9.3)	1.949 (3.9)	3,870 (7.7)
2014-2016	34,646 (13.9)	3,801 (11.0)	1,678 (4.8)	3,193 (9.2)
**Infant characteristics**				

Sex					
Male	126,988 (51.1)	10,924 (8.6)	4,168 (3.3)	9,132 (7.2)
Female	121,630 (48.9)	10,461 (8.6)	3,971 (3.3)	8,838 (7.3)
Gestation at childbirth (weeks)				
<31.9	545 (1.3)	58 (10.6)	23 (4.2)	48 (8.8)
32-36.9	2,361 (5.7)	243 (10.3)	114 (4.8)	200 (8.5)
>37	38,474 (93.0)	2,698 (7.0)	1,079 (2.80)	2,207 (5.7)
Unknown	207,238	18,386 (8.9)	6,923 (3.3)	15,515 (7.5)
Birth weight (Kg)				
<1.50	24 (0.4)	..	..	..
1.50-2.49	233 (4.1)	22 (9.4)	14 (6.0)	16 (6.9)
2.50-2.99	838 (14.8)	72 (8.6)	34 (4.1)	60 (7.2)
3.00-3.49	2,022 (35.8)	155 (7.7)	71 (3.5)	120 (5.9)
3.50-3.99	1,798 (31.8)	121 (6.7)	48 (2.7)	101 (5.6)
>4.00	739 (13.1)	52 (7.0)	15 (2.0)	45 (6.1)
Unknown	242,964	20,959 (8.6)	7,954 (3.3)	17,625 (7.3)
Apgar at 1 minute				
0-3	588 (1.7)	65 (11.1)	30 (5.1)	60 (10.2)
4-6	2,043 (6.0)	184 (9.0)	88 (4.3)	144 (7.1)
7-10	31,702 (92.3)	2,338 (7.4)	945 (3.0)	1,885 (6.0)
Unknown	214,285	18,798 (8.8)	7,076 (3.3)	15,881 (7.4)
Apgar at 5 minutes				
0-3	65 (0.2)	..	..	..
4-6	377 (1.1)	35 (9.3)	16 (4.2)	29 (7.7)
7-10	33,665 (98.7)	2,522 (7.5)	1,040 (3.1)	2,030 (6.0)
Unknown	214,511	18,822 (8.8)	7,080 (3.3)	15,905 (7.4)
Special care at birth				
NICU	6,477 (2.6)	804 (12.4)	284 (4.5)	702 (10.8)
Unknown	242,141 (97.4)	20,581 (8.5)	7,845 (3.2)	17,268 (7.1)

Table B: Infant healthcare use for sensitive definition of perinatal anxiety, comparing those with and without maternal perinatal anxiety, antenatal anxiety and postnatal anxiety

Characteristics	Rate of consultations per person-year (95% Confidence Intervals)			
	No anxiety	Perinatal anxiety	Antenatal anxiety	Postnatal anxiety
Overall	8.6 (8.6-8.6)	9.6 (9.6-9.6)	9.6 (9.6-9.7)	9.6 (9.6-9.6)
Maternal age (years)				
15-19	8.8 (8.7-8.8)	9.4 (9.2-9.5)	9.7 (9.4-10.0)	9.4 (9.2-9.6)
20-24	9.0 (8.9-9.0)	9.7 (9.6-9.8)	10.0 (9.8-10.1)	9.8 (9.7-9.9)
25-29	8.9 (8.9-8.9)	9.9 (9.8-10.0)	10.0 (9.8-10.1)	9.9 (9.8-10.0)
30-34	8.7 (8.6-8.7)	9.7 (9.6-9.8)	9.7 (9.6-9.8)	9.7 (9.6-9.8)
35-39	8.3 (8.3-8.3)	9.2 (9.1-9.3)	9.1 (9.0-9.3)	9.2 (9.1-9.3)
40-44	8.0 (8.0-8.1)	9.1 (8.9-9.2)	9.3 (9.0-9.5)	9.0 (8.8-9.1)
45-49	8.2 (8.0-8.4)	8.8 (8.2-9.5)	7.9 (7.1-8.9)	8.7 (8.0-9.5)
Townsend Score quintile				

1-least deprived	8.8 (8.7-8.8)	9.9 (9.8-10.0)	9.8 (9.6-10.0)	9.9 (9.8-10.0)
2	8.6 (8.6-8.7)	9.7 (9.6-9.8)	9.8 (9.6-10.0)	9.7 (9.6-9.8)
3	8.7 (8.6-8.7)	9.6 (9.5-9.7)	9.7 (9.5-9.8)	9.5 (9.4-9.6)
4	8.8 (8.8-8.8)	9.7 (9.6-9.8)	9.7 (9.6-9.9)	9.7 (9.6-9.8)
5-most deprived	8.7 (8.7-8.7)	9.5 (9.4-9.6)	9.5 (9.4-9.7)	9.5 (9.4-9.6)
Unknown	8.3 (8.2-8.3)	9.2 (9.1-9.3)	9.3 (9.2-9.5)	9.2 (9.1-9.3)
History of anxiety				
Recent	9.3 (9.1-9.3)	9.6 (9.6-9.7)	9.6 (9.5-9.6)	9.7 (9.6-9.8)
Previous	9.2 (9.1-9.3)	9.9 (9.8-10.0)	10.1 (9.8-10.4)	9.9 (9.7-10.0)
None	8.6 (8.6-8.6)	9.5 (9.5-9.6)	9.7 (9.6-9.9)	9.5 (9.4-9.6)
History of Depression				
Recent	9.1 (9.0-9.1)	9.5 (9.5-9.6)	9.5 (9.4-9.6)	9.6 (9.5-9.7)
Previous	9.1 (9.0-9.2)	9.9 (9.8-10.1)	10.1 (9.8-10.4)	10.0 (9.8-10.1)
None	8.6 (8.6-8.6)	9.6 (9.5-9.6)	9.9 (9.7-10.0)	9.6 (9.5-9.6)
Mode of delivery				
Vaginal delivery	8.7 (8.7-8.7)	9.6 (9.5-9.6)	9.7 (9.5-9.8)	9.5 (9.5-9.6)
Instrumental	9.2 (9.1-8.8)	9.7 (9.6-9.9)	9.7 (9.4-9.9)	9.8 (9.7-10.0)
Caesarean	8.8 (8.8-8.8)	9.6 (9.5-9.6)	9.9 (9.7-10.1)	9.7 (9.6-9.8)
Unknown	8.5 (8.5-8.5)	9.6 (9.5-9.6)	9.6 (9.5-9.7)	9.6 (9.5-9.6)
Year group				
1998-2001*	9.2 (9.1-9.2)	10.3 (10.2-10.5)	10.6 (10.3-10.9)	10.2 (10.1-10.4)
2002-2004	8.8 (8.8-8.9)	9.8 (9.7-9.9)	10.1 (9.9-10.3)	9.8 (9.7-9.9)
2005-2007	8.9 (8.9-8.9)	9.8 (9.7-10.0)	10.0 (9.8-10.2)	9.8 (9.7-9.9)
2008-2010	8.6 (8.6-8.7)	9.4 (9.4-9.5)	9.5 (9.3-9.6)	9.4 (9.3-9.5)
2011-2013	8.5 (8.5-8.5)	9.6 (9.5-9.7)	9.7 (9.6-9.8)	9.6 (9.5-9.7)
2014-2016	7.8 (7.8-7.8)	9.1 (9.0-9.1)	9.0 (8.8-9.0)	9.1 (9.0-9.2)
**Infant characteristics**				
Sex				
Male	8.9 (8.9-8.9)	9.8 (9.8-9.9)	9.9 (9.8-10.0)	9.8 (9.8-9.9)
Female	8.3 (8.3-8.3)	9.3 (9.3-9.4)	9.4 (9.3-9.5)	9.4 (9.3-9.4)
Gestation at childbirth (weeks)				
<31.9	9.8 (9.5-10.1)	9.6 (8.8-10.5)	9.6 (8.3-11.0)	9.6 (8.8-10.6)
32-36.9	10.3 (10.1-10.4)	12.1 (11.7-12.6)	12.1 (11.5-12.8)	12.2 (11.7-12.7)
>37	9.4 (9.4-9.5)	10.7 (10.6-10.8)	10.8 (10.6-11.0)	10.9 (10.7-11.0)
Unknown	8.5 (8.4-8.5)	9.4 (9.4-9.4)	9.4 (9.4-9.5)	9.4 (9.3-9.4)
Birth weight (Kg)				
<1.50	10.7 (9.3-12.3)	..	..	..
1.50-2.49	9.7 (9.3-10.1)	12.5 (11.0-14.0)	13.2 (11.4-15.3)	12.8 (11.1-14.7)
2.50-2.99	9.0 (8.8-9.3)	9.8 (9.1)	10.4 (9.4-11.6)	9.7 (8.9-10.5)
3.00-3.49	9.1 (8.9-9.2)	9.2 (8.7-9.7)	8.8 (8.1-9.5)	9.1 (8.6-9.7)
3.50-3.99	9.1 (8.9-9.2)	9.5 (9.0-10.1)	8.9 (8.0-9.7)	9.6 (9.0-10.2)
>4.00	8.8 (8.5-9.0)	9.2 (8.4-10.1)	8.5 (7.1-10.1)	9.2 (8.4-10.2)
Unknown	8.6 (8.6-8.6)	9.6 (9.6-9.6)	8.7 (8.7-8.7)	8.6 (8.6-8.7)
Apgar at 1 minute				
0-3	10.0 (9.7-10.3)	..	..	..
4-6	10.0 (9.9-10.2)	11.4 (10.9-11.9)	12.0 (11.3-12.8)	11.5 (10.9-12.0)
7-10	9.4 (9.4-9.4)	10.8 (10.6-10.9)	10.8 (10.6-11.1)	10.9 (10.7-11.0)
Unknown	8.5 (8.5-8.5)	9.4 (9.4-9.5)	8.6 (8.5-8.6)	8.5 (8.5-8.5)

Apgar at 5 minutes				
0-3	10.0 (9.2-10.8)	8.3 (6.2-11.0)	10.0 (6.7-14.3)	8.3 (6.2-11.0)
4-6	10.1 (9.8-10.4)	11.1 (10.0-12.2)	10.1 (8.5-11.8)	11.0 (9.8-12.3)
7-10	9.5 (9.4-9.5)	10.8 (10.7-11.0)	11.0 (10.8-11.2)	11.0 (10.8-11.1)
Unknown	8.5 (8.5-8.5)	9.4 (9.4-9.5)	8.6 (8.5-8.6)	8.5 (8.5-8.5)
Special care at birth				
NICU	9.0 (9.0-9.1)	9.5 (9.3-9.8)	9.6 (9.2-9.9)	9.6 (9.3-9.8)
Unknown	8.6 (8.6-8.6)	9.6 (9.6-9.6)	9.7 (9.6-9.7)	9.6 (9.6-9.6)

Table C: Infant preventative healthcare uptake and model analysis sensitive definition of perinatal anxiety, comparing those with and without maternal antenatal anxiety

**Outcomes**	**5-in-1 vaccination**		**6-8 week infant check***	
	**Yes**	**No**	**Yes**	**No**
**n (%)**				
No Perinatal anxiety	215,179 (94.7)	12,054 (5.3)	175,416 (82.8)	36,555 (17.3)
Perinatal anxiety	20,422 (95.5)	963 (4.5)	16,534 (81.1)	3,850 (18.9)

**Model analysis**	**Unadjusted: OR (95% CI)**	**Adjusted[1]: OR (95% CI)**	**Unadjusted: OR (95% CI)**	**Adjusted[1]: OR (95% CI)**
No Perinatal anxiety	1	1	1	1
Perinatal anxiety	1.21 (1.12-1.31)	1.40 (1.29-1.51)	0.91 (0.86-0.97)	0.92 (0.86-0.97)

*Footnotes: OR: Odds Ration;CI: Confidence Interval; *analysis includes records from 2001 onwards only (n=232,355 for summary statistics and n=195,539 for model analysis); 1- adjusted for age, deprivation and year; Practice is included as a random effects term in all models.*
